# Biological rhythms in COVID-19 vaccine effectiveness in an observational cohort study of 1.5 million patients

**DOI:** 10.1172/JCI167339

**Published:** 2023-06-01

**Authors:** Guy Hazan, Or A. Duek, Hillel Alapi, Huram Mok, Alex Ganninger, Elaine Ostendorf, Carrie Gierasch, Gabriel Chodick, David Greenberg, Jeffrey A. Haspel

**Affiliations:** 1 Division of Pulmonary and Critical Care Medicine, Department of Internal Medicine, Washington University School of Medicine, St. Louis, Missouri, USA.; 2 Department of Pediatrics, Soroka University Medical Center, Beer-Sheva, Israel.; 3 Research and Innovation Center, Saban Children’s Hospital, Beer-Sheva, Israel.; 4 Department of Psychiatry, Yale University School of Medicine, New Haven, Connecticut, USA.; 5 Department of Epidemiology, Biostatistics and Community Health Sciences, Faculty of Health Sciences, Ben-Gurion University of the Negev, Beer-Sheva, Israel.; 6 Maccabitech Institute for Research and Innovation, Maccabi Healthcare Services, Tel Aviv, Israel.; 7 Sackler Faculty of Medicine, Tel Aviv University, Tel Aviv, Israel.; 8 Pediatric Infectious Disease Unit, Soroka University Medical Center, Beer-Sheva, Israel.; 9 Faculty of Health Sciences, Ben-Gurion University of the Negev, Beer-Sheva, Israel.

**Keywords:** COVID-19, Vaccines, Statistics

## Abstract

**BACKGROUND:**

Circadian rhythms are evident in basic immune processes, but it is unclear if rhythms exist in clinical endpoints like vaccine protection. Here, we examined associations between COVID-19 vaccination timing and effectiveness.

**METHODS:**

We retrospectively analyzed a large Israeli cohort with timestamped COVID-19 vaccinations (*n* = 1,515,754 patients over 12 years old, 99.2% receiving BNT162b2). Endpoints included COVID-19 breakthrough infection and COVID-19–associated emergency department visits and hospitalizations. Our main comparison was among patients vaccinated during morning (800–1159 hours), afternoon (1200–1559 hours), or evening hours (1600–1959 hours). We employed Cox regression to adjust for differences in age, sex, and comorbidities.

**RESULTS:**

Breakthrough infections differed based on vaccination time, with lowest the rates associated with late morning to early afternoon and highest rates associated with evening vaccination. Vaccination timing remained significant after adjustment for patient age, sex, and comorbidities. Results were consistent in patients who received the basic 2-dose series and who received booster doses. The relationship between COVID-19 immunization time and breakthrough infections was sinusoidal, consistent with a biological rhythm that modifies vaccine effectiveness by 8.6%–25%. The benefits of daytime vaccination were concentrated in younger (<20 years old) and older patients (>50 years old). COVID-19–related hospitalizations varied significantly with the timing of the second booster dose, an intervention reserved for older and immunosuppressed patients (HR = 0.64, morning vs. evening; 95% CI, 0.43–0.97; *P* = 0.038).

**CONCLUSION:**

We report a significant association between the time of COVID-19 vaccination and its effectiveness. This has implications for mass vaccination programs.

**FUNDING:**

NIH.

## Graphical abstract

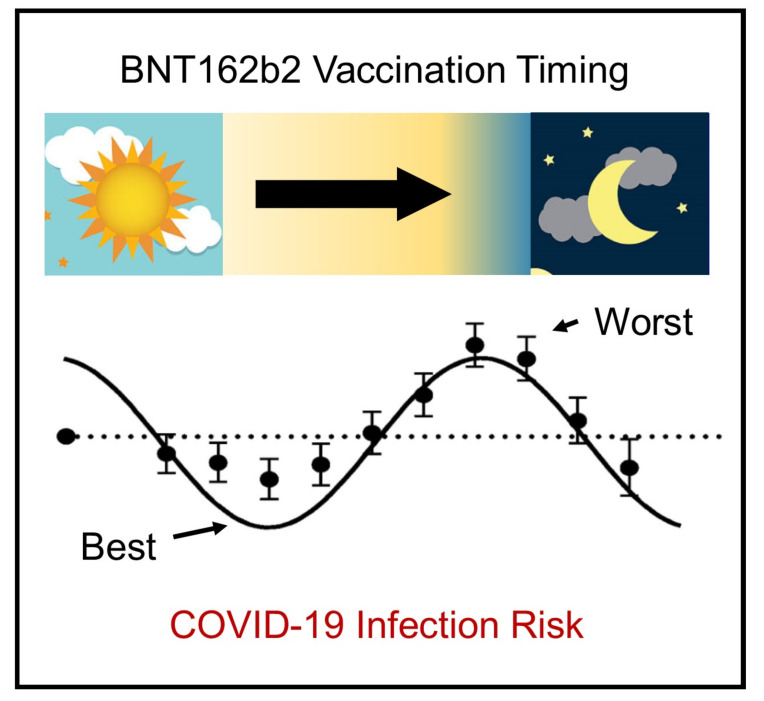

## Introduction

Circadian rhythms are daily oscillations in biological function that enable organisms to align their physiology to the day-night cycle (1, 2). These rhythms emanate from a genetically encoded molecular clock that regulates gene expression and thereby organizes cellular functions into daily cycles (1, 2). Among the activities organized by the circadian system are basic immune processes (3–5), including the innate inflammatory response (6–12), cellular egress from the bone marrow (13), leukocyte trafficking to organs and lymph nodes (14–16), and T cell responses to antigen (17, 18). Some theorize that the circadian rhythms in humans should translate, directly or indirectly, into preferable times of day to vaccinate patients (1, 2, 19–23). However, clinical studies are equivocal on whether rhythms exist in vaccine responses, including those against SARS-CoV-2, the cause of COVID-19 (24–35). While important, prior studies reported surrogate markers of immunogenicity like antibody titers rather than clinical outcomes and had limited sample sizes. Here, we examined how the timing of COVID-19 vaccination relates to clinical protection using large-scale observational data.

## Results

To examine how the timing of COVID-19 immunization relates to clinical effectiveness we analyzed a large cohort of patients enrolled in Maccabi Healthcare Services (MHS), a major Israeli health maintenance organization (HMO). A strength of the MHS data is that prior studies have used it and other similar sources to measure the real-world effectiveness of COVID-19 vaccines (36–39). However, these studies did not consider the time of immunization as a factor.

**Figure 1:**
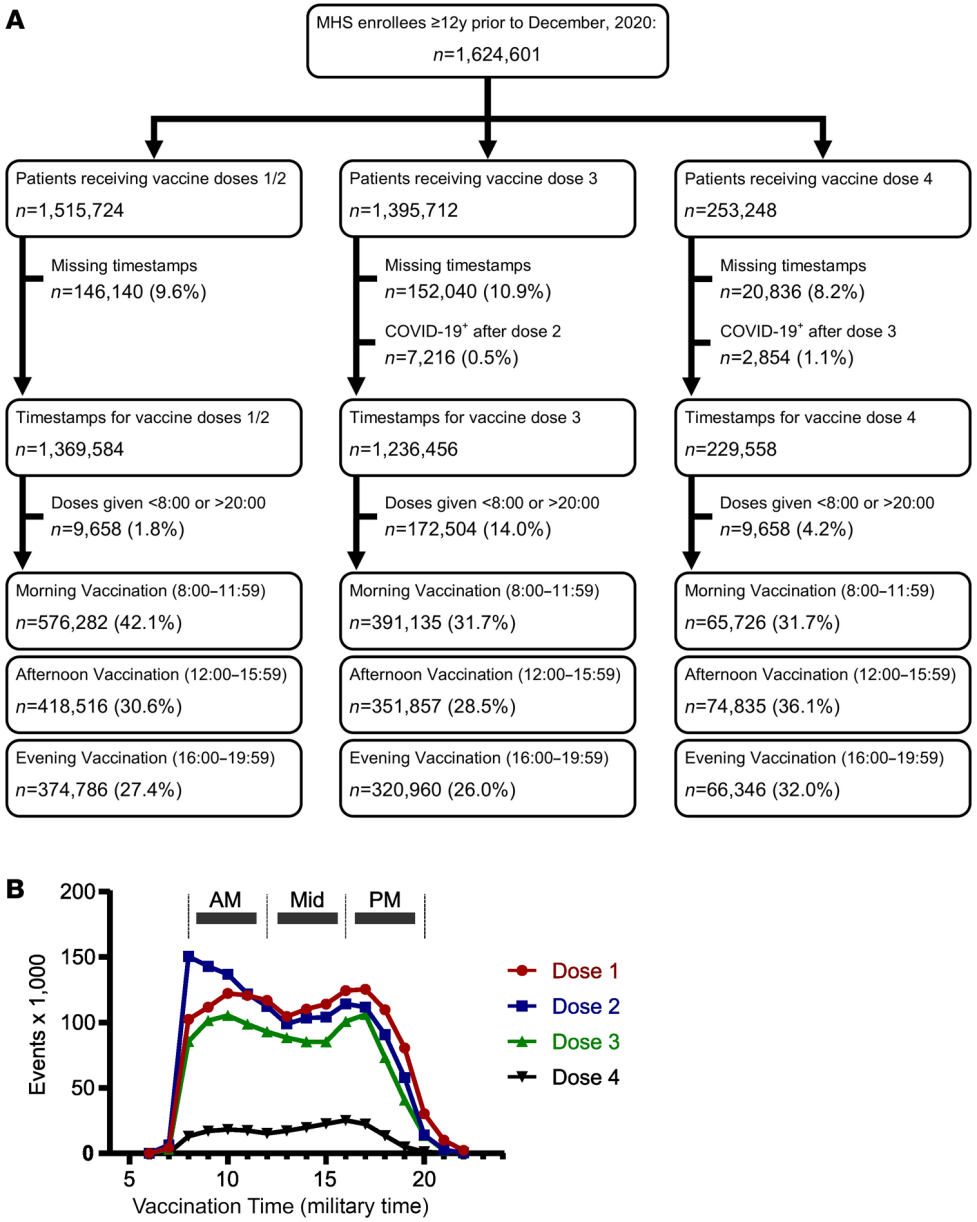
SARS-CoV2 vaccine timing across the day. (**A**) The patient inclusion flowchart. (**B**) The distribution of COVID-19 vaccine administration times, binned by hour of the day. Red circles indicate dose 1; blue squares indicate dose 2; green triangles indicate dose 3 (the first booster dose); and black inverted triangles indicate dose 4 (second booster dose). Time intervals used to compare the effects of morning (AM), afternoon (Mid), and evening (PM) vaccine dosing on effectiveness are denoted by horizontal bars.

Of approximately 2.6 million MHS participants, 1,515,574 had timestamps recorded for at least 1 immunization (Figure 1A and Supplemental Figure 1; supplemental material available online with this article; https://doi.org/10.1172/JCI167339DS1). Our study population almost exclusively received the Pfizer-produced BNT162b2 vaccine (99.2%, *n* = 1,503,599), with a minority receiving the Moderna mRNA-1273 product (0.74%, *n* = 11,220). Most patients received immunizations within a 12-hour time span stretching from 800 to 2000 hours (Figure 1B). Based on this distribution, we began by comparing COVID-19 breakthrough infections in patients who received their immunization in the morning (800–1159 hours, *n* = 552,423), afternoon (1200–1359 hours, *n* = 418,516), or evening (1600–1959 hours, *n* = 374,786). For the initial 2-dose immunization series, we based our analysis on the timing of dose 2, which completes the intervention. As a group, patients vaccinated in the morning were older and had more comorbidities than at other times, although the magnitude of these differences were small (Table 1). Over the study period, the cohort incurred 278,488 COVID-19–positive tests. There were also 4,501 COVID-19–associated emergency department (ED) visits and 3,824 COVID-19–associated hospitalizations, defined as occurring within –7 to +7 days of a COVID-19–positive test (see Methods).

**Figure 2:**
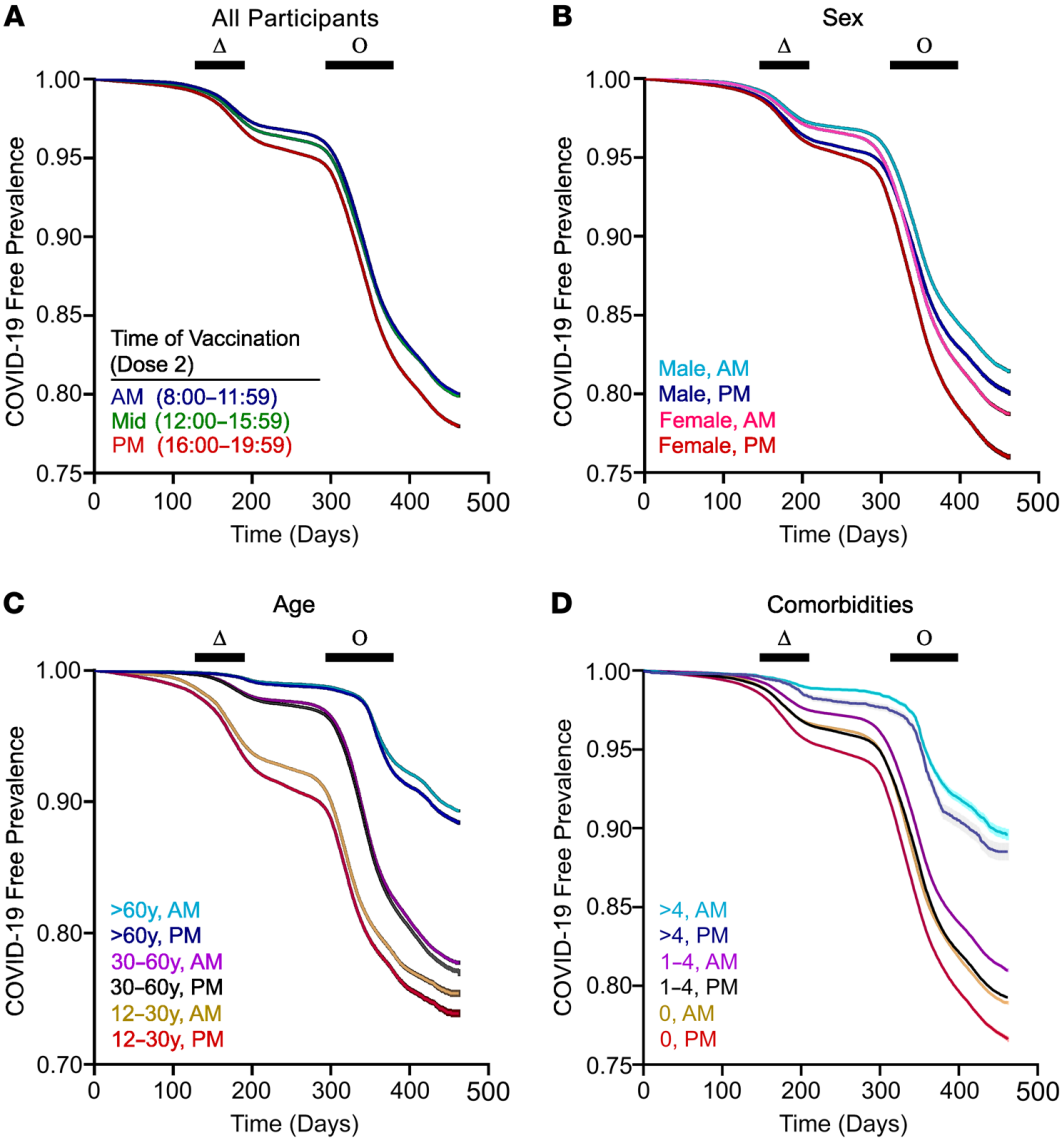
Association of COVID-19 breakthrough infections with vaccination timing. (**A**) Infection-free survival in patients receiving the initial vaccine series between 800 and 1159 hours (morning [AM]), 1200 and 1559 hours (afternoon [Mid]), and 1600 and 1959 hours (evening [PM]). (**B**–**D**) COVID-19–free survival in patients stratified by (**B**) sex, (**C**) age, and (**D**) number of medical comorbidities. Shading around the lines represent 95% CIs. Waves of COVID-19 infection caused by the Delta (Δ) and Omicron (Ο) SARS-CoV-2 variants based on Israeli Ministry of Health data are indicated. For clarity, **B**–**D** display only the AM and PM groups.

Patients immunized against COVID-19 in the morning and afternoon had less frequent breakthrough infections than those vaccinated in the evening, with the groups diverging prior to the wave of infections caused by the Delta variant (Figure 2A). The difference remained significant after stratification by sex, age, and total number of comorbidities (Figure 2, B–D). A subgroup analysis of patients who received both doses 1 and 2 exclusively in the morning, midday, or evening yielded similar results (Supplemental Figure 2). Counterintuitively, factors like older age that predict worse COVID-19 clinical outcomes were associated with fewer breakthrough infections, a finding also noted elsewhere (37, 39). We suggest that this reflects greater adherence to COVID-19 precautions in older or more vulnerable individuals (for example, mask use and social distancing), leading to less viral exposure. Even so, morning vaccination remained superior to evening vaccination in these patients (Figure 2, C and D).

We considered whether patients receiving vaccinations at different times of day might have different baseline infectious risks due to unmeasured variables in our data, for example occupation or household size. To this end, we scrutinized COVID-19 infections in the first 14 days after the first immunization, a time frame prior to the onset of full vaccine protection. During this period there were 7.0, 8.1, and 3.6 COVID-19 infections per 100,000 patients in the morning, afternoon, and evening groups, respectively (*P* = 0.008, morning vs. evening, and *P* = 0.245, morning vs. afternoon, permutation test). This pattern is the opposite of the effectiveness signal seen after vaccination takes effect, favoring morning or afternoon dosing (Figure 2A). Because patients vaccinated in the morning are older and have more comorbidities (Table 1), adjustment for these demographic variables would further widen the difference between morning and evening groups. Thus, baseline infection risk does not explain why morning and afternoon vaccinations were associated with fewer breakthrough infections after immunization.

We also considered if our comparison groups might differ in their readiness to undergo COVID-19 testing, thus confounding results. However, the morning and evening vaccination groups had equivalent numbers of COVID-19 tests (2.28 vs. 2.29 tests per capita, respectively) and a similar distribution of testing times around the clock (Supplemental Figure 3A). We did observe reduced positivity rates in COVID-19 tests obtained in the early morning, similar to one recent report (40), but this pattern was independent of vaccination timing (Supplemental Figure 3B). Overall, univariate analysis suggested a clinical advantage to dosing COVID-19 vaccines in the morning or afternoon in terms of fewer breakthrough infections. These results are unlikely to reflect differences in the baseline velocity of COVID-19 infection, diagnostic testing patterns, or diurnal variations in COVID-19 detection.

**Figure 3:**
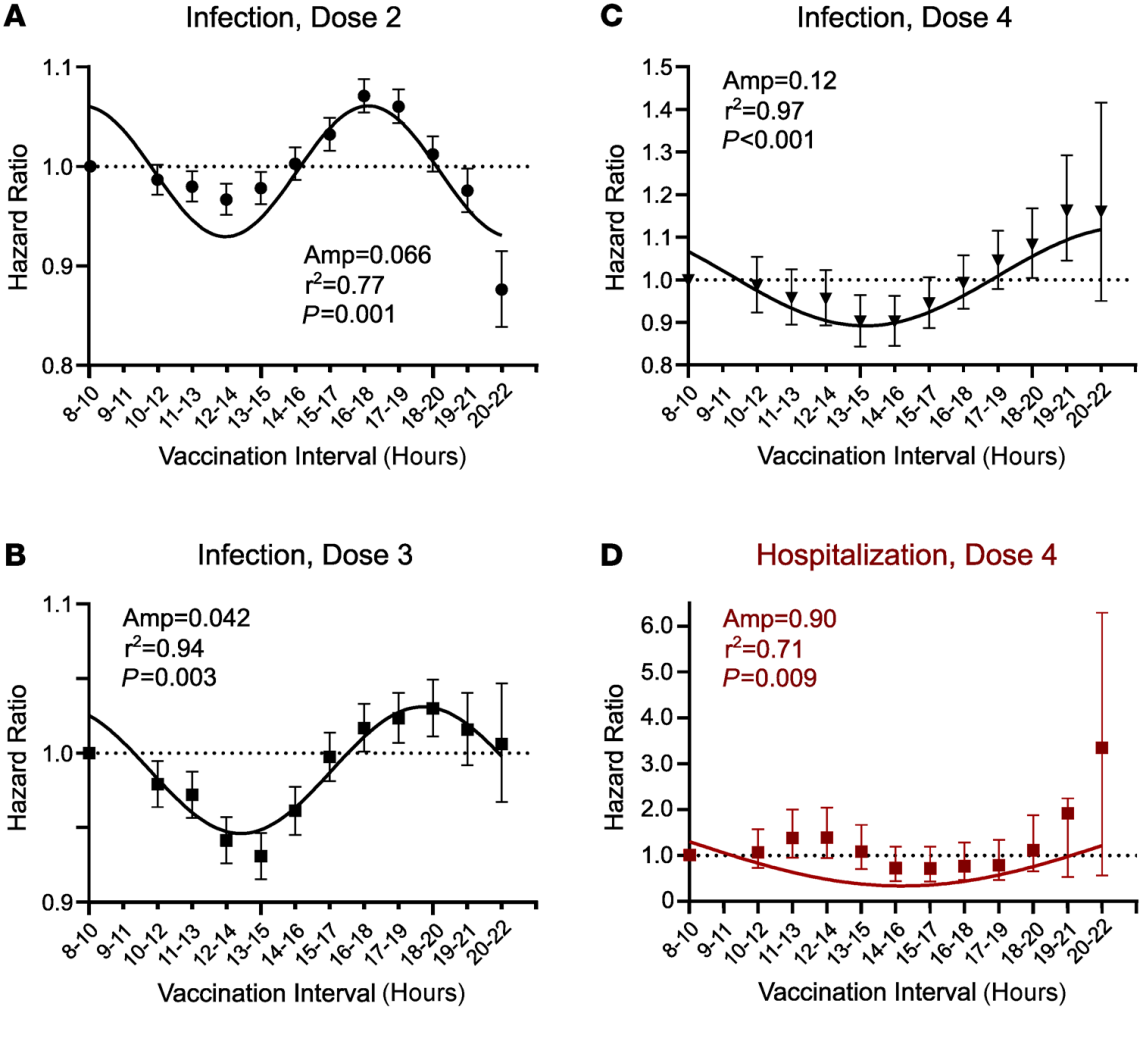
Variation in adjusted COVID-19 breakthrough infection and hospitalization risk as a function vaccine timing. Data points represent adjusted HRs ± 95% CI, relative to dosing between 800 and 959 hours, which serves as a nonoverlapping index bin for this analysis. Best-fit sinusoidal trend lines (black lines), amplitudes (Amp), goodness of fit (r^2^), and METACYCLE-generated *P* values for periodicity are depicted within each graph. (**A**–**C**) HRs for breakthrough infections (black symbols) based on the timing of vaccine (**A**) dose 2, (**B**) dose 3, and (**C**) dose 4. (**D**) HRs for COVID-19–associated hospitalizations based on the timing of dose 4. We define COVID-19–associated ED visits or hospitalization as occurring between –7 to +7 days of a COVID-19–positive test (see Methods). For patient demographic breakdown, sample sizes, and a tabular presentation of these data, see Supplemental Tables 10, 11, and 13. For the complete METACYCLE output, see Supplemental Table 14.

Using Cox regression to adjust for age, sex, comorbidities, and the protection afforded by COVID-19 boosters, we mapped the relationship between vaccination time and breakthrough infections (Figure 3). Across the time span when most COVID-19 vaccinations were given, the HR for breakthrough infections was lowest between late morning and early afternoon and highest for evening vaccination times (Figure 3, A–C). Stratifying participants into morning, midday, and evening vaccination groups produced similar conclusions, with optimal vaccine timing straddling the morning and midday bins (Table 2). Two alternative statistical approaches to Cox modeling, logistic regression and bootstrap analysis, also produced similar conclusions (Supplemental Tables 4 and 5). The relationship between vaccination time and HR for breakthrough infection was sinusoidal, with period durations ranging from 7.4 to 16.7 hours, depending on the vaccine dose and the method used to assess rhythm parameters (Figure 3, A–C, and Supplemental Table 14). We estimated maximum peak-to-trough change in the HR for breakthrough infection as 0.13 for doses 1 and 2, 0.086 for dose 3, and 0.25 for dose 4 (Figure 3, A–C). In our cohort, this translates to a number needed to treat (NNT) range of 18.7 to 54.5 by study end if patients were moved from the least favorable to most favorable COVID-19 vaccination times. It also corresponds to a relative change of vaccine effectiveness of 8.6%–25%. For doses 2 and 3, time of vaccination did not significantly associate with COVID-19–related ED visits or hospitalizations, nor was there a sinusoidal trend in HRs across the day (Table 2 and Supplemental Figure 6, A and B). However, for dose 4 (the second booster dose), vaccination timing did significantly associate with COVID-19 hospitalizations, favoring morning immunization (HR = 0.64; morning vs. evening, 0.43–0.97 95% CI; *P* = 0.038), and there was a nonsignificant trend for ED visits (Table 2). In both cases, the relationship between dose 4 timing and outcomes was sinusoidal (Figure 3D, Supplemental Figure 6C, and Supplemental Table 12) and resembled that of breakthrough infection risk (Figure 3C). Thus, COVID-19 vaccination timing had a significant association with effectiveness, as defined by breakthrough infection and, in the case of the second booster shot, hospitalization. The sinusoidal trend suggests a biological rhythm in COVID-19 vaccine effectiveness based on time of administration.

**Figure 4:**
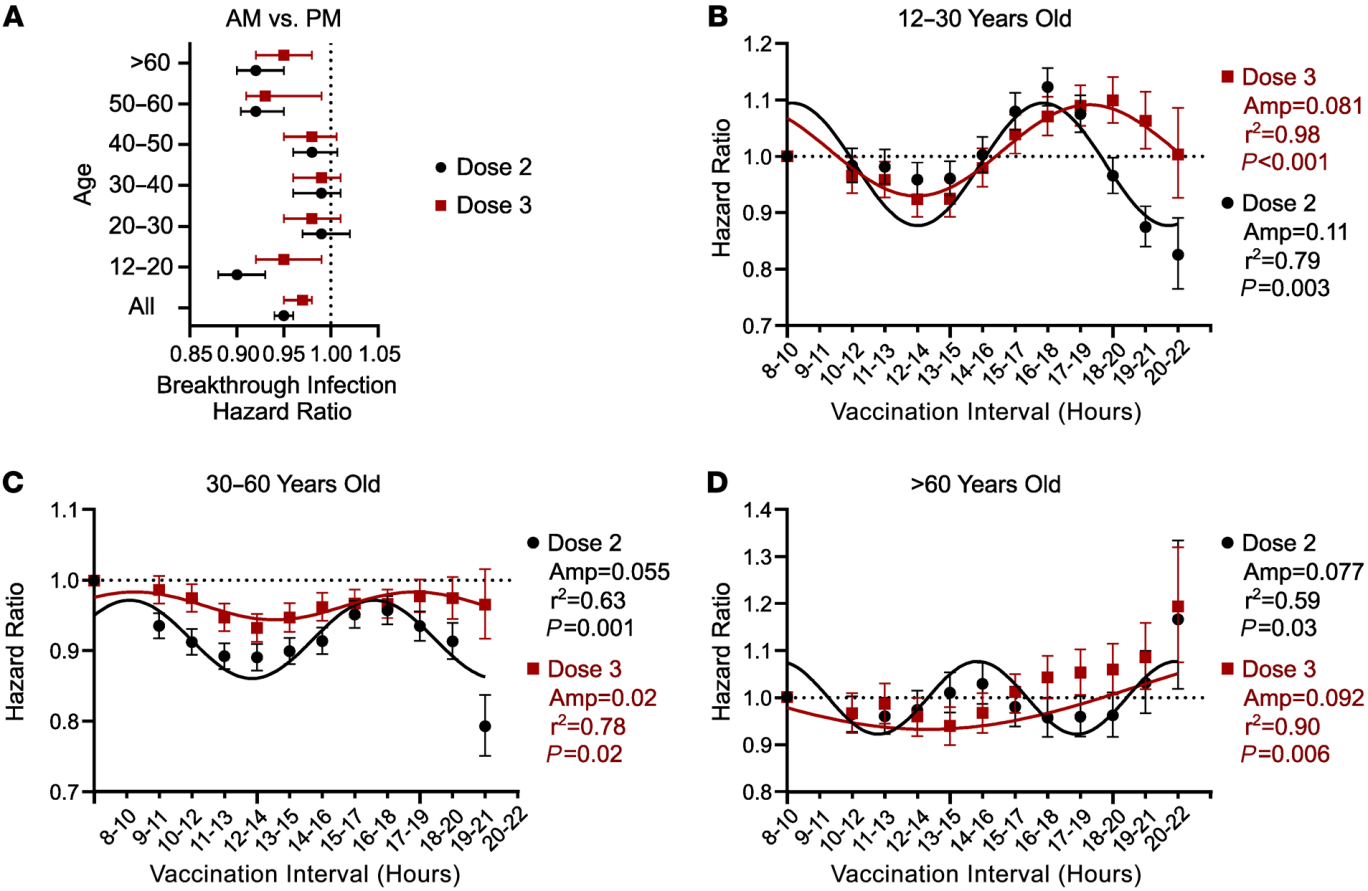
Effect of patient age on SARS-CoV-2 vaccine rhythms. (**A**) HRs ± 95% CI for breakthrough COVID-19 infection based on time of vaccination (AM versus PM) and age range. Values to the left of the dotted line favor morning vaccination (800–1159 hours), and values to the left favor evening vaccination (1600–1959 hours). Black symbols indicate that timing of dose 2 was considered. Red symbols indicate that timing of dose 3 was considered. For a comparison of afternoon vs. evening vaccination times as function of age, see Supplemental Figure 7 and Supplemental Table 16. (**B**–**D**) Comparative vaccine effectiveness around the clock relative to dosing between 800 and 959 hours (HR ± 95% CI). Best-fit sinusoidal trend lines (black lines), amplitudes (Amp), goodness of fit (r^2^), and METACYCLE-generated *P* values for periodicity are depicted within each graph. Black, timing of dose 2 is considered. Red, timing of dose 3 is considered. (**B**) Ages 12–30 years old. (**C**) Ages 30–60 years old. (**D**) Over 60 years old. For sample sizes and tabular presentation of these data, see Supplemental Tables 15 and 17–19. For the complete METACYCLE output, see Supplemental Table 14.

During the study period, the second booster was restricted to patients over 60 and those with a history of immunosuppression. As such we examined how chronological age interacts with immunization timing with regards to vaccine effectiveness (Figure 4). We found that the benefits of morning vaccination were concentrated in younger (<20 years old) and older (>50 years old) individuals (Figure 4A and Supplemental Figure 7). Older age correlated with a shift in the peak HR to later hours (Figure 3C and Figure 4, B–D). These data suggest a strategy for leveraging biological rhythms to optimize COVID-19 vaccine effectiveness in a population, where younger patients and the elderly would be prioritized for immunizations in the late morning to early afternoon.

## Discussion

Our data indicate a significant association between the time of COVID-19 vaccination and its clinical effectiveness in terms of breakthrough infection and hospitalization. While the effect of vaccination timing is additive on top of an effective vaccine, it is an easily modifiable factor that when extended over millions of immunizations can amount to a large aggregate benefit. The relationship between vaccination timing and infection risk is sinusoidal, suggesting a biological rhythm in vaccine effectiveness consistent with circadian regulation of underlying immune processes. Studies indicate that fundamental immune processes exhibit circadian and ultradian rhythms in controlled model systems (6–12, 17, 18). Our study contributes for the first time to our knowledge a “bottom line,” real-world estimate of clinical protection afforded by optimal vaccination timing.

While breakthrough COVID-19 infections varied with vaccination timing for all doses studied, ED presentations did not achieve statistical significance, and hospitalizations showed a time effect for only the second booster (dose 4). Of note, our statistical power for detecting associations with ED presentations or hospitalizations was much lower, as they were infrequent in our cohort (60.8 and 71.5 positive COVID tests per ED visit or hospitalization, respectively). This fact accentuates the value of analyzing large cohorts for detecting diurnal effects on infrequent but clinically important outcomes. Another explanation is that patients presenting with pneumonia severe enough to merit emergency care are more likely to have disrupted circadian rhythms at baseline (41), and thus time of vaccination might be less biologically pertinent to them. Regardless, minimizing COVID-19 infections of any severity is desirable. Asymptomatic patients can still infect others, and even mild symptoms can complicate the management of chronic conditions. Moreover, reducing hospitalizations in older patients is important, as they are at increased risk for poor outcomes.

We observed that the time of vaccination was most salient for patients at the extremes of age (<20 and >50 years old). Potentially this reflects changes to the circadian system and immune experience with aging (Figure 4, B–D) (41, 42). Alternatively, working age adults may be more likely to engage in night shift work or other nighttime activities that could mask circadian effects at a population level. Regardless, the observation is useful because it suggests that prioritizing young patients and the elderly for morning-to-midday immunization is enough to improve the effectiveness of mass vaccinations against COVID-19. Such a prioritization might extend to other vaccinations like influenza, given studies suggesting that morning influenza immunizations are beneficial specifically in the elderly (30, 33). We suggest that a timed vaccination approach for specific age ranges is practical and warrants prospective study. Such a prospective trial could combine actigraphy and longitudinal viral surveillance with immunogenicity biomarkers, including neutralizing antibody titers, pathogen-specific memory B and T cells, and innate immune memory responses in peripheral blood mononuclear cells. That way, the trial would deliver a clinical conclusion on efficacy while helping to elucidate potential mechanisms. It would also help to resolve conflicting studies about time-of-day variations in SARS-CoV-2 antibody titers (28, 31, 32).

Human populations have less consistent behavioral patterns than other organisms, making it challenging to observe how circadian rhythms affect medical interventions in real-world settings. This is because individuals lead diverse lifestyles, are routinely exposed to light at night, and engage in activities like night shift work that alter the phase of the circadian clock (19, 42). Our ability to detect rhythms in COVID-19 vaccine effectiveness in this study is due to the unique circumstances of the COVID-19 pandemic and the Israeli response to the pandemic. This includes a population-level sample of participants that is two orders of magnitude larger than prior cohorts, a concerted intervention largely consisting of a single vaccine product (BNT162b2), a population highly motivated to report COVID-19 infection, substantial clinical follow-up (>1 year), and, finally, a high level of data integration within the Israeli healthcare system. Our data do not directly address whether rhythms in BNT162b2 effectiveness extend to other COVID-19 vaccine products or to vaccinations against other pathogens. However, it is worth noting that the plurality of studies that do report diurnal rhythms in vaccine immunogenicity found morning-to-afternoon dosing to be optimal (25, 29, 30). Therefore, our data may have implications for diseases beyond COVID-19.

Our study has several strengths. Biological rhythms in vaccination are an understudied topic, with only 12 published studies in patients, including 6 focused on COVID-19 (24–35). These prior studies differ on whether optimal vaccination times exist, what times of day produce optimal vaccine responses, and whether the effect is specific to certain patient subsets. While important, earlier studies had relatively low sample sizes, variable design, short-term follow-up, differences in vaccine type, and, most notably, a focus on markers of vaccine immunogenicity like antibody titers as endpoints rather than clinical protection. For BNT162b2, antibody titers vary by up to two orders of magnitude in healthy individuals, making this a noisy readout for observing circadian effects (43). Moreover, the dose-response relationship between spike antibody titers and COVID-19 vaccine efficacy appears weak (44). Our study avoids this pitfall by directly examining how immunization time of day relates to real-world BNT162b2 vaccine protection. Other strengths include a cohort an order of magnitude larger than all prior studies combined, longer clinical follow-up (>16 months), and orthogonal validation of conclusions using independent statistical approaches. Recent studies reveal time of day to be a significant factor in natural coronavirus infection beyond vaccine responses, including SARS-CoV-2 viral entry (45), mucosal barrier function (46), and, as we confirm here, SARS-CoV-2 positivity rates (Supplemental Figure 3B) (40). Thus, biological rhythms are evident at multiple levels of COVID-19 infection, and our data demonstrate that they extend to a key medical intervention.

Our analysis also has limitations. As with any observational study, patients were not randomly assigned to specific vaccination times, and demographic differences between groups can bias results. We attempted to compensate for this by adjusting for variables known to affect COVID-19 infection rates and complications (39, 47, 48), assessing the role of unmeasured variables, and using independent statistical models. Nevertheless, there may be sources of bias missed by our methods. There were also a limited number of ED and hospitalization events in the study population to support conclusions related to this specific outcome.

Our data set lacks comprehensive viral genotyping, and so we cannot determine whether time of vaccination influences breakthrough infections to different extents across variants. However, given rhythms were evident with vaccine boosters given while different viral variants were dominant our findings are likely to be broadly applicable.

Because vaccinations were not provided around the clock in our population, we cannot be certain whether the observed rhythms in vaccine effectiveness are diurnal (i.e., 24-hour cycles) or ultradian in nature (<24-hour cycles) as suggested by periodicity analysis (Figures 3 and 4 and Supplemental Table 14). Regardless, both diurnal and ultradian rhythms are compatible with upstream circadian clock influence (18, 49).

Israel is located at 31 degrees latitude and experiences warmer winters and less seasonal variation in photoperiod than more northerly or southerly latitudes. It is conceivable that viral transmission might be faster in areas with colder air temperature (50), thereby obscuring circadian influences in vaccine effectiveness in those regions. However, COVID-19 incidence did not vary by latitude within the US, which lies between the 20th and 66th parallels (51). Moreover, we observed a similar diurnal pattern in SARS-CoV-2 positivity rates as a cohort from the US (40). Thus, it is likely that rhythms in BNT162b2 vaccine effectiveness extend to other countries.

Our cohort is relatively ethnically homogenous (91% identifying as Israeli Jewish), and validation in more diverse populations would be valuable. That said, data indicate that BNT162b2 exhibits similar efficacy across ethnic boundaries (52), suggesting that demographics are likely not a limitation to generalizability.

Our results reflect the cumulative behavior of our cohort, and individuals with acutely disrupted circadian rhythms due to shift work, jet lag, or sleep deprivation might react differently to immunization timing. Research suggests these patients are more vulnerable to COVID-19 infection, and future research should focus on vaccination strategies specific to this subgroup (53).

Finally, our data cannot determine by themselves the mechanism by which time of day regulates COVID-19 vaccine protection. Based on observations in model systems, it is tempting to speculate that circadian clock regulation of innate inflammation and T cell activation might explain the oscillations in vaccine effectiveness reported here (12, 17, 18). Further research should explore these possibilities.

In summary, circadian rhythms represent a fundamental property of living things that regulate the immune system at a basic biological level. This study provides the first estimate to our knowledge of how far this core process extends into an important clinical realization of immune function: vaccine effectiveness. Our data suggest a way of leveraging biological rhythms to optimize immunizations against SARS-CoV-2 variants using reformulated vaccines and potentially vaccines against other pathogens.

## Methods

### Study design.

This retrospective cohort study analyzes database records from MHS in Israel. MHS is the second largest state-mandated, not-for-profit, HMO in Israel with over 2.6 million members, constituting one-quarter of the country’s population. MHS maintains extensive medical, demographic, and anthropometric information linked to a nation-wide electronic medical record servicing the entire Israeli civilian population. The MHS database captures all clinical encounters, diagnoses, medications, and laboratory data for its participants anywhere within the country regardless of setting, including all clinical activity and diagnostic testing related to COVID-19.

Our analysis used the target-trial framework (54) and focused on the time of day vaccines were given for the initial series (doses 1 and 2), the first booster dose (dose 3), or the second booster (dose 4). We conceptualized each vaccine encounter as a separate clinical question as to the optimal time of vaccine effectiveness.

### Setting.

In December 2020, Israel launched a national immunization campaign against COVID-19 consisting almost exclusively of the Pfizer BNT162b2 vaccine series delivered in 2 doses 3–4 weeks apart (Supplemental Figure 1). In July and September 2021, the Israeli Ministry of Health initiated third (first booster) and fourth (second booster) dose campaigns, respectively. Vaccines and point-of-care COVID-19 testing were provided free of charge to all citizens. During the study period, 96.2% of point-of-care tests were PCR based, and antigen-based point-of-care testing was only briefly significant during the wave of Omicron variant (B.1.1.529) infections (maximum monthly prevalence 10.8% of all tests). A monthly breakdown of point-of-care testing by detection modality is provided in Supplemental Figure 4. At-home antigen test kits were available for purchase (retail cost range, NIS 32–100, or $11–$32 per kit), and in fall 2021 20 home kits were provided free to families with school-aged children to support the back-to-school effort (Supplemental Figure 4, arrow). However, the Israeli Ministry of Health required a documented positive test prior to quarantine and a negative test after quarantine before issuing a certificate of recovery to patients throughout the study period (https://corona.health.gov.il/en/confirmed-cases-and-patients/cases-recovered/). Thus, censoring of COVID-19 infection is unlikely in our cohort as there was strong incentive to obtain a laboratory diagnosis even if initial testing occurred at home.

### Participants.

Among 1,624,601 MHS participants over 12 years of age 1,594,180 (98.13%) received the Pfizer BNT162b2 vaccine, 29,253 (1.8%) received the Moderna mRNA-1273 vaccine, 437 (0.03%) received the AstraZeneca ChAdOx1-S product, 400 (0.02%) received Sputnik V, 232 (0.01%) received the Jansen Jcovden vaccine, and 46 (0.003%) received the Sinovac CoronaVac product; for 53 patients (0.003%) the vaccine identity was unspecified.

We analyzed data from December 19, 2020 (the first day of vaccine administration in the study population), to April 25, 2022 (the last day of data extraction). This time span encompasses two spikes in COVID infection dominated by the Delta (B.1.617.2) and Omicron (B.1.1.529) SARS-CoV-2 variants (Supplemental Figure 1, black and blue bars).

We extracted data from all MHS members aged 12 years and older who received at least 1 dose of SARS-CoV-2 vaccine and had joined MHS prior to February 2020 and, therefore, had a complete medical history on file. For analysis of each vaccine dose, we excluded patients who had a documented SARS-CoV-2–positive test prior to the date of vaccination (Figure 1A). We further excluded patients with missing timestamps (Figure 1A). 81.1% of MHS members identified as Jewish, 6% identified as orthodox Jews, 5.8% as Arabs, and 6.1% as former Soviet Union residents.

### Variables.

We analyzed deidentified patient-level data extracted from MHS electronic records. Continuous variables included age at immunization, time and date of vaccine administration, and body mass index. Dichotomous variables included sex and comorbidities linked to COVID-19 severity (47, 48). For variable definitions, see Supplemental Tables 1 and 2.

### Data sources/measurements.

All data used for analysis was extracted from the MHS database. The primary outcome was COVID-19 infection as defined by positive SARS-CoV-2 PCR or antigen test performed at any official site. Infections in the first 7 days following vaccination were excluded from the analysis, since patients during this interval are not considered to have a complete immunologic response to vaccination. Secondary outcomes were ED visits and hospitalizations associated with COVID-19 infection, defined as encounters within a period –7 to +7 days after a documented COVID-19–positive test.

### Bias.

Typically, a Schoenfeld’s global test is used to validate the assumption of Cox proportional hazards. However, the large sample sizes of our study present a challenge to this approach because this leads to overpowering (55). We found evidence of this in our data by applying the Schoenfeld’s global test on stratified data with smaller sample sizes (Supplemental Table 3), demonstrating reduction in test significance compared with the complete data set. Therefore, we used two alternate approaches to orthogonally validate results from our Cox model as recommended (55): multivariate logistic regression (Supplemental Table 4) and bootstrap multivariate analysis (Supplemental Table 5). For logistic regression, we applied the same multivariate Cox regression model, with the exception that follow-up time was not included. The reason for this modification was that our goal with logistic regression was to validate the trend rather than the effect size estimated by the Cox model and that follow-up times were not materially different between vaccination groups (Table 1). For bootstrap analysis, we sampled the entire cohort for 2,000 iterations with replacement calculating HRs and CIs for the same multivariate regression model. Results from both approaches showed similar trends as our multiple Cox regression model, supporting this approach for analyzing the data. As additional support, a plot of scaled Schoenfeld residuals for the variable time of vaccination demonstrated stable proportional HRs across the study period (Supplemental Figure 5) (56).

### Study size.

We used expansive inclusion criteria covering all vaccinated patients without prior documented COVID-19 infection in the MHS database that had demographic information and timestamped vaccinations.

### Data availability.

Deidentified data will be made available with a transfer agreement.

### Statistics.

An initial descriptive analysis included calculations of single variable distribution, central tendency, and dispersion for all data in Table 1. We stratified vaccine timing in 4-hour bins for comparison: 800–1159 hours (morning), 1200–1559 hours (afternoon), and 1600–1959 hours (evening) (Figure 1B). For analysis of the initial vaccine series (dose 1 and 2), day 0 in our analysis was individually defined for each patient as 7 days after the receipt of vaccine dose 2 as described previously (36). Given that the first 2 doses of BNT162b2 constitute a single intervention, we combined these doses into one analytic model. Our main comparison for the initial vaccine series (doses 1 and 2) was based on the timing of dose 2 only. In support of this, a sensitivity analysis demonstrated that dose 2 timing was more important than dose 1 in modifying breakthrough infection risk for the initial vaccine series (Supplemental Table 6). For analysis of the booster doses (doses 3 and 4), day 0 was defined as 7 days after the immunization was given. Note that only the time of booster administration was considered, as we regarded boosters as separate medical interventions to augment waning COVID-19 immunity.

Because one independent variable (booster status) differed over time, univariate and multivariate survival analyses were performed with time-dependent covariates (57, 58). Kaplan–Meier analysis with a log-rank test was used for the univariate analysis. For analyzing the initial vaccine series based on the timing of dose 2, we employed a Cox proportional–hazards regression model with random mixed effects to estimate the association between vaccine administration time of day and study endpoints. For analyzing the first booster dose (dose 3), we used a Cox proportional–hazards regression model and adjusted for dose 4 as a time-dependent covariant. For analyzing time of vaccination of the second booster (dose 4), we applied a Cox proportional–hazards regression model without time-dependent covariates. Cox modeling was previously applied to Israeli HMO data for analyzing associations between COVID-19 immunization and clinical endpoints over extended periods (39). Our choice of variables to include in the Cox model was based on previously published associations between age, comorbidities, and COVID-19 endpoints of infection or disease severity (39, 47, 48). Then, we interrogated violation of Cox assumptions for the variables of interest by computing a Schoenfeld global test (see *Bias* above). Variables that were associated with outcomes measured (breakthrough COVID-19 infection or COVID-19–related ED visits) with a *P* < 0.1 in the univariate comparison were included in the multivariate model. For the multivariate analysis, we used goodness-of-fit parameters (Akaike Information Criterion [AIC] = –2 log likelihood+ 2*p*, where *p* denotes the number of estimated parameters in this equation with a backward elimination approach). To simplify the modeling of breakthrough events after the initial immunization series (doses 1 and 2), we censored events in the subset of patients receiving the second booster (dose 4) after the date it was administered. For modeling events after doses 3 and 4, there was no censoring. We treated the first COVID-19 infection as a terminal event. As such, recurrent infections did not factor into our analysis.

We calculated NNT as described previously (59): NNT = (Sc^ΔHR^ – Sc)^–1^, where Sc is event-free survival in the reference group, and ΔHR is the HR at the most preferable minus the least preferable vaccination time. We estimated Sc as 0.75 at study end based on the survival curve generated for univariate analysis (Figure 2A) and ΔHR as the peak-to-trough difference in HR for breakthrough infection across the day (Figures 3 and 4). We estimated the change in vaccine effectiveness based on moving vaccinations from the best to worst time of day as 1-HR as described previously (39).

### Time bin sensitivity analysis.

To examine how the choice of time bins affected conclusions from our Cox regression model, we generated HRs comparing the 800- to 959-hour time bin to successive 2-hour intervals across the day, incrementing by 1 hour with each iteration. Note that to avoid overlap with the index bin, HRs were generated from 1000 hours in this analysis. Our hypothesis was that a biological rhythm in vaccine effectiveness should produce a sinusoidal trend in HRs as a function of vaccination time. To statistically evaluate the trend in HRs across the day for periodicity we used METACYCLE, an algorithm commonly used for circadian rhythm detection in gene expression data (60). The COSOPT algorithm (61) was used to generate best-fit sinusoidal trendlines for data display. We observed very high goodness of fit with this approach (r^2^ range, 0.77–0.97, Figure 3), supporting the use of a sinusoidal model. To avoid forced fitting these data, we programmed COSOPT and METACYCLE to select the best fitting periodic function across a range of cycle lengths (4–24 hours) as described previously (61).

We performed all statistical analyses using R version 3.5.0. Code for all analyses can be found in the Supplemental Methods. A *P* value of less than 0.05 was used to indicate significance in all analyses.

### Study approval.

The study was approved by the MHS Ethical Committee. All data were deidentified prior to analysis. Because this was a retrospective study, patient informed consent was not required.

## Author contributions

GH and JAH conceived the study. GH, OAD, HM, AG, EO, CG, DG, and JH contributed to the study design. Data were extracted by HA, GH, and OAD. GC oversaw the data gathering. All authors contributed to the data analysis. GH and JAH wrote the initial manuscript. All authors vouch for the data and analysis. All authors participated in the editing and critical review of the manuscript and decided to submit it for publication.

## Supplementary Material

Supplemental data

Trial reporting checklists

ICMJE disclosure forms

Supplemental data set 1

## Figures and Tables

**Table 2:**
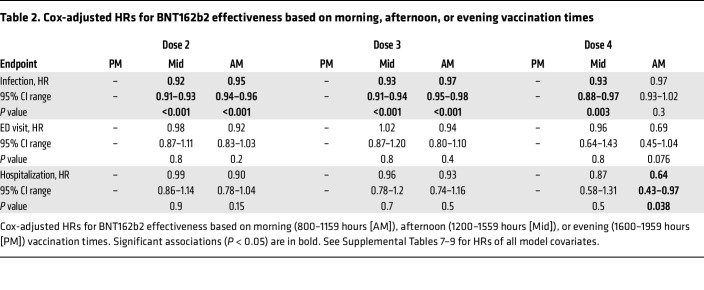
Cox-adjusted HRs for BNT162b2 effectiveness based on morning, afternoon, or evening vaccination times

**Table 1:**
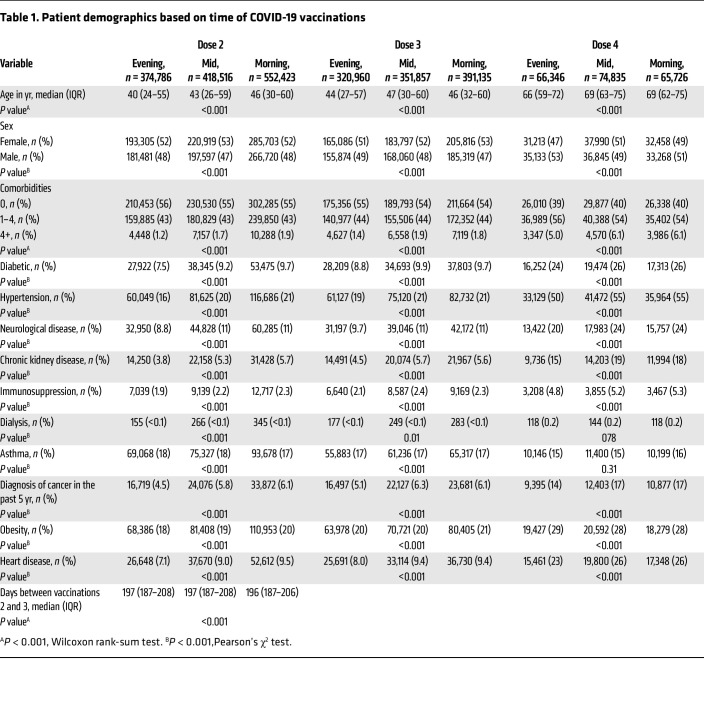
Patient demographics based on time of COVID-19 vaccinations

## Version 1 04/25/2023

In-Press Preview

## Version 2 06/01/2023

Electronic publication
