# Supplementary material for: Biological rhythms in COVID-19 vaccine effectiveness in an observational cohort study of 1.5 million patients
Source: J Clin Invest. 2023 Jun 1;133(11):e167339. doi: 10.1172/JCI167339 (PMC10231992; doi:10.1172/JCI167339)
Supplement: Supplemental data [file jci-133-167339-s153.pdf]

SUPPLEMENTAL FIGURES AND TABLES

BIOLOGICAL RHYTHMS IN COVID-19 VACCINE EFFECTIVENESS, AN OBSERVATIONAL COHORT STUDY OF 1.5 MILLION PATIENTS

Guy Hazan, M.D., Ph.D.[1-3], Or A. Duek, PhD[4,5], Hillel Alapi, B.A.[6], Huram Mok, MD[1], Alex Ganninger[1], Elaine Ostendorf[1], Carrie Gierasch, B.S.[1], Gabriel Chodick, Ph.D.[6,7], David Greenberg, M.D.[8,9], and Jeffrey A. Haspel, M.D., Ph.D.[1*].

[1]Division of Pulmonary and Critical Care Medicine, Department of Internal Medicine, Washington University School of Medicine, St. Louis, MO, USA.

[2]Department of Pediatrics, Soroka University Medical Center, Beer-Sheva, Israel.

[3]Research and Innovation Center, Saban Childrens Hospital, Beer-Sheva, Israel.

[4]Department of Psychiatry, Yale University School of Medicine, New Haven, CT, USA.

[5]Department of Epidemiology, Biostatistics and Community Health Sciences, Faculty of Health Sciences, Ben-Gurion University of the Negev, Beer-Sheva, Israel

[6]Maccabitech Institute for Research and Innovation, Maccabi Healthcare Services, Tel Aviv, Israel.

[7]Sackler Faculty of Medicine, Tel Aviv University, Tel Aviv, Israel.

[8]The Pediatric Infectious Disease Unit, Soroka University Medical Center, Beer-Sheva, Israel.

[9]Faculty of Health Sciences, Ben-Gurion University of the Negev, Beer-Sheva, Israel.

*Corresponding author contact information: Jeffrey Haspel, Division of Pulmonary and Critical Care Medicine, Department of Internal Medicine, Washington University School of Medicine, Campus Box 8052, 660 South Euclid Avenue, St. Louis, MO, 63110, USA. Email: jhaspel@wustl.edu

Supplemental Figure 1

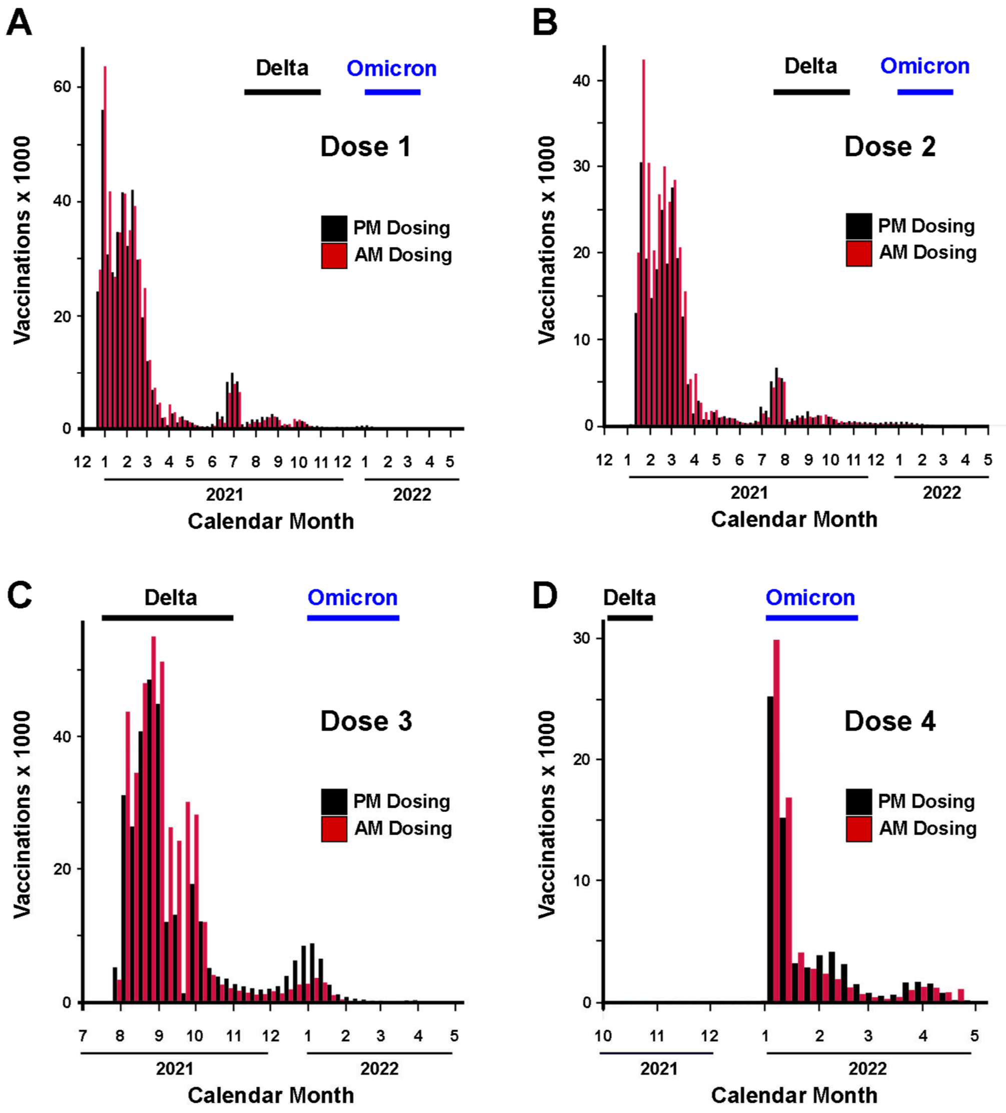

Supplemental Figure 1. COVID-19 vaccination schedule in the MHS cohort. Bars represent the number of vaccines given between 8:00-11:59 (AM dosing, red bars) and 16:00-19:59 (PM dosing, black bars). Calendar months are depicted numerically with 1 representing January and 12 representing December. Spikes in infection rates within the Israeli population caused by the delta and omicron SARS-CoV-2 variants as reported by the Israeli Ministry of Health (MOH) are indicated by black and blue horizontal bars, respectively. **(A-D)** show vaccine doses over time for the first (**A**), second (**B**), first booster (third dose, **C**), and second booster doses (fourth dose, **D**).

Supplemental Figure 2

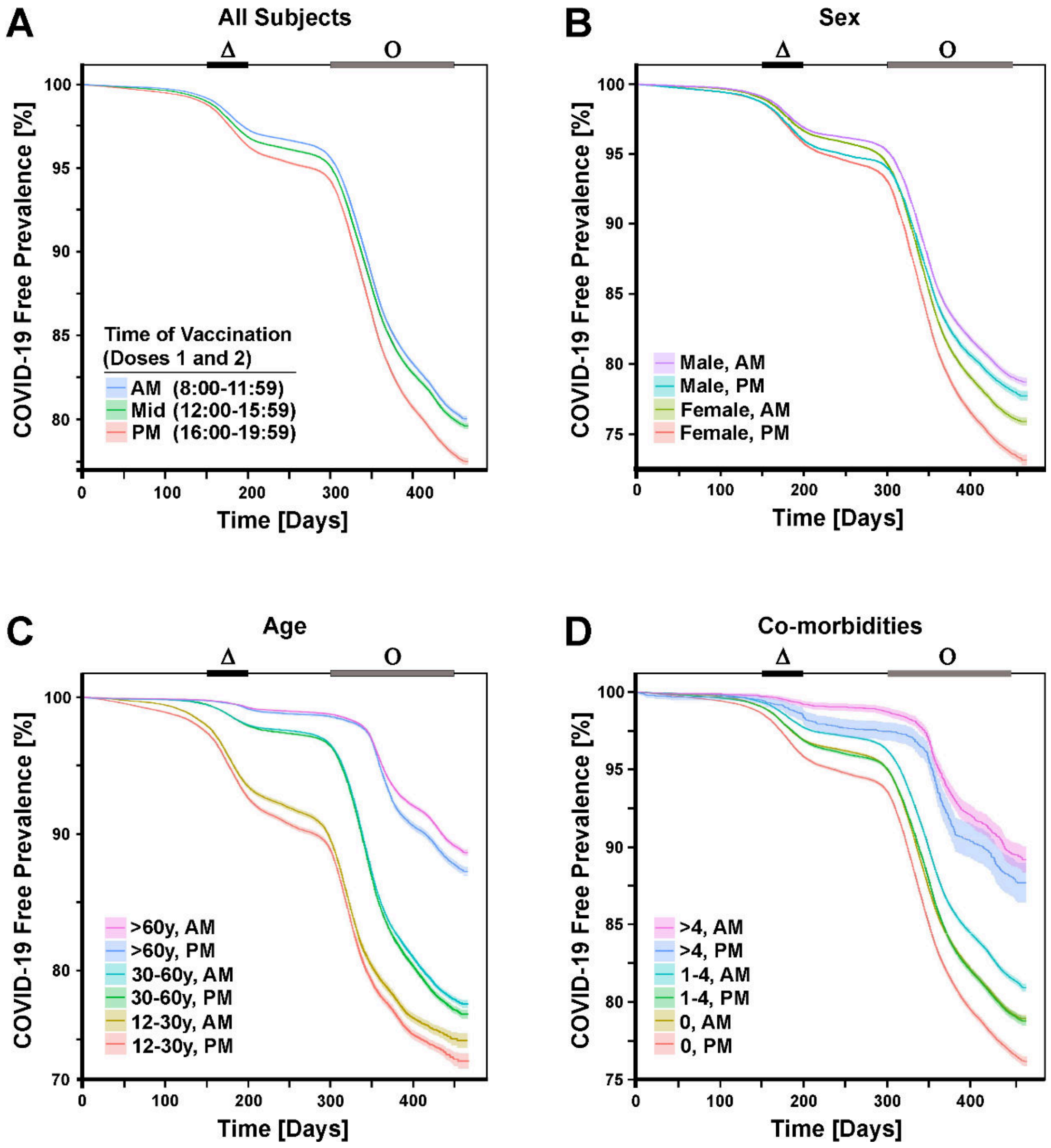

Supplemental Figure 2. Association of COVID-19 breakthrough infections with vaccination timing. (**A**) shows infection-free survival in patients receiving the first two COVID-19 vaccine inoculations between 8:00-11:59 hours (morning or AM, n=313,844), 12:00-15:59 hours (afternoon or Mid, n=292,348), and 16:00-19:59 hours (evening or PM, n=236,348). (**B-D**) show COVID-19-free survival in patients stratified by sex (**B**), age (**C**), and number of medical co-morbidities (D). Shading around the lines represent 95% confidence intervals (CI). Waves of COVID-19 infection caused by the delta (Δ) and omicron (O) SARS-CoV-2 variants based on Israeli Ministry of Health (MOH) data are indicated by black and grey horizontal bars, respectively. For clarity, panels B-D display only the AM and PM groups.

Supplemental Figure 3

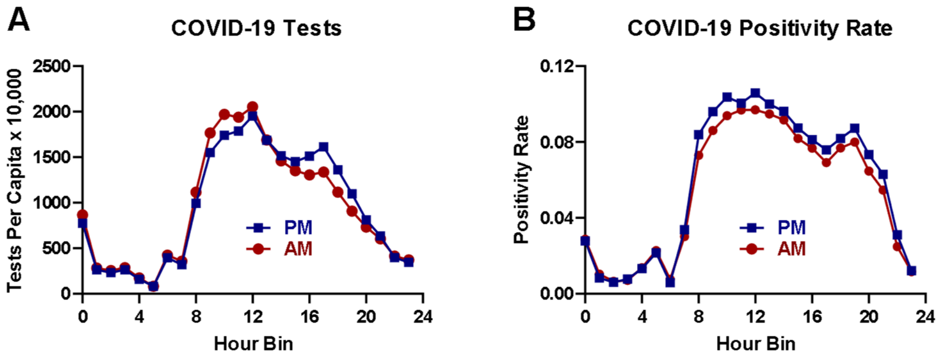

Supplemental Figure 3. Distribution of COVID-19 positivity across the day as a function of COVID-19 vaccine timing. **(A and B)** show the distribution of total COVID tests (**A**) and positivity rates (**B**) across the day for patients receiving the initial COVID-19 vaccine series (doses 1 and 2). AM, patients immunized between 8:00-11:59 (red symbols). PM, patients immunized between 16:00-19:59 (blue symbols). For sample sizes and tabular presentation of these data see **Supplemental Table 20**.

Supplemental Figure 4

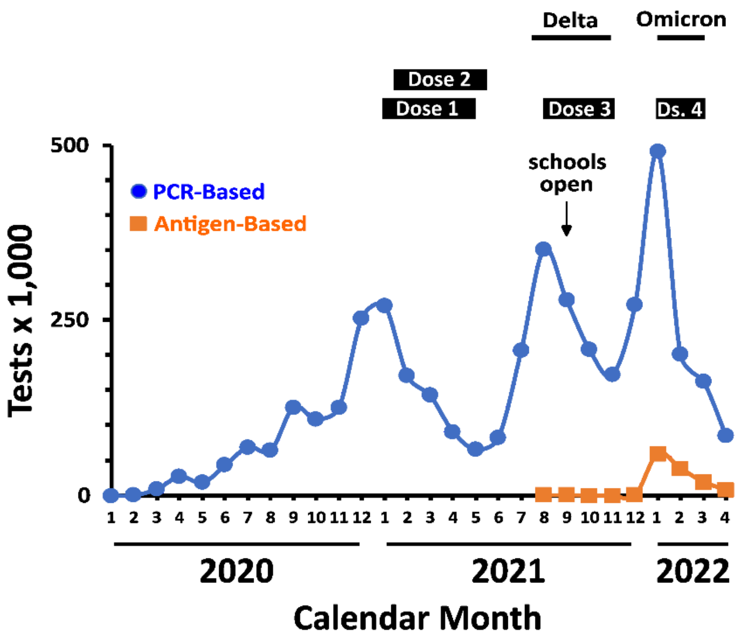

Supplemental Figure 4. COVID-19 testing in the MHS cohort. Lines represent monthly COVID-19 point-of-care tests in the MHS cohort from January 2020 to the end of the study period. Blue circles, PCR-based tests. Orange squares, antigen-based tests. Calendar months are depicted numerically with 1 representing January and 12 representing December. Spikes in infection rates within the Israeli population caused by the delta and omicron SARS-CoV-2 variants as reported by the Israeli Ministry of Health (MOH) are indicated by indicated by thin horizontal lines. Thick lines represent peak intervals of vaccine administration for the initial vaccine series (doses 1 and 2), the first booster (dose 3), and the second booster (dose 4) which restricted to patients over 60. In class schooling resumed in Israel on September 1, 2021 (arrow).

Supplemental Figure 5

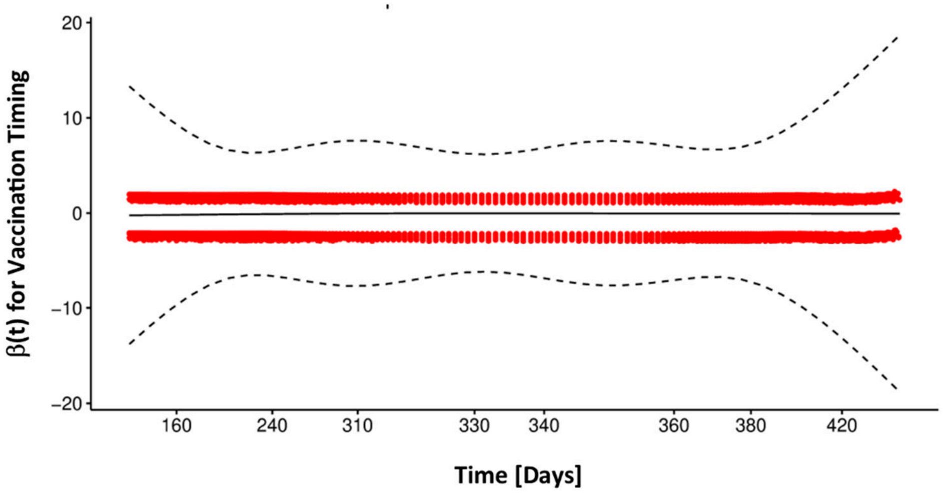

Supplemental Figure 5. Plot of scaled Schoenfeld residuals for vaccination timing over the study period. Dashed lines, 95% CI. For discussion of Cox proportional hazards assumption, see the "Bias" section in Methods.

Supplemental Figure 6

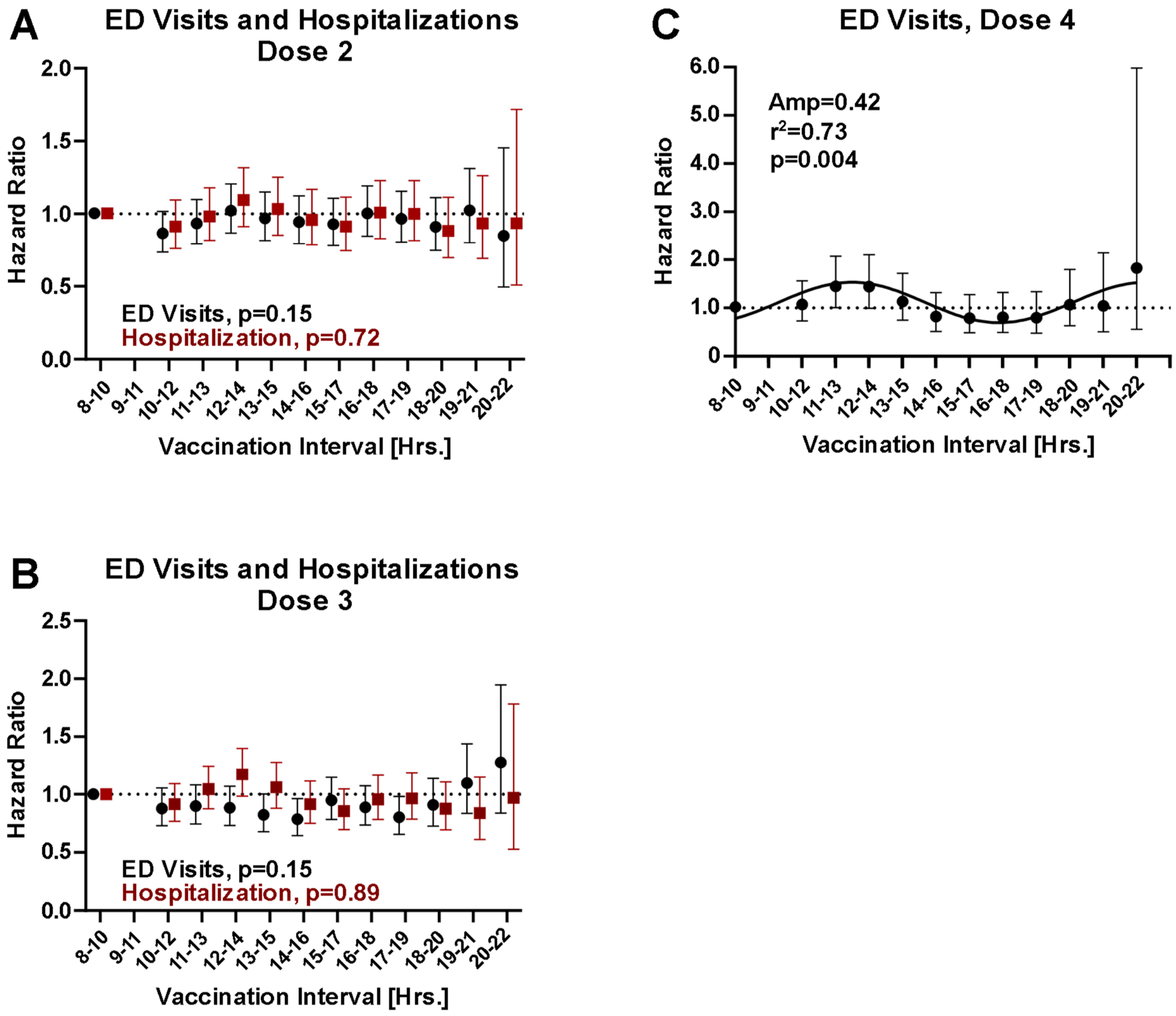

Supplemental Figure 6. Variation in adjusted risk of COVID-19 associated ED visits and hospitalization as a function vaccine timing. Data points represent adjusted hazard ratios (HR) ± 95% CI relative to dosing between 8:00-9:59 AM, which serves a non-overlapping index bin for this analysis. Best-fit sinusoidal trend lines (black lines), amplitudes (Amp), period duration (Per), goodness of fit (r^2), and METACYCLE generated p-values for periodicity are depicted within each graph. (**A and B**) show HRs for ED visits (black symbols) and hospitalization (red symbols) based on the timing of vaccine dose two (**A**), dose three (**B**). (**D**) shows HRs for COVID-19-associated ED visits based on the timing of Dose 4. We define COVID-19 associated ED visits or hospitalization as occurring between -7 to +7 days of a COVID-19 positive test (see Methods). For patient demographic breakdown, sample sizes, and a tabular presentation of these data see **Supplemental Table 8 and 9**. For the complete METACYCLE output see **Supplemental Table 14**.

Supplemental Figure 7

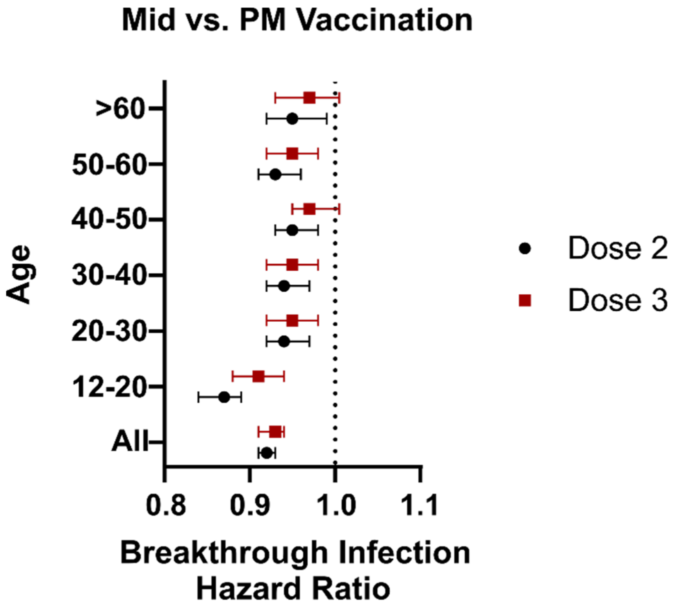

Supplemental Figure 7. Breakthrough infection risk as a function of age, comparing afternoon (Mid, 12:00-15:59) to evening (PM, 16:00-19:59) vaccination times. Data points represent adjusted hazard ratios (HR) ± 95% CI. Values to the left of the dotted line favor afternoon vaccination, and values to the left favor evening vaccination. Black, timing of dose two is considered. Red, timing of dose three is considered. For a tabular presentation of these data see **Supplemental Table 16**.

Supplemental Table 1: Outcome variable definitions. Note, we report all outcome variables extracted for this project regardless of whether they entered into the analysis reported by this manuscript.

Variable	Values	Definitions	Timing
Documented SARS-CoV-2 Infection	0/1	A PCR confirmed infection.	The date and time-of-day of a specimen collection that was found to be positive in a PCR test.<br><br>If the PCR test was done after the beginning of a hospitalization flagged as a COVID-19 hospitalization, the infection date was set to the beginning of the hospitalization.
Documented SARS-CoV-2 Infection		A PCR: Cycle threshold number.	
Asymptomatic SARS-CoV-2 Infection	0/1	A PCR-confirmed infection with no report of symptoms during referral and during initial physician questioning.	The date set for theSARS-CoV-2 infection outcome.
COVID-19 (symptomatic SARS-CoV-2 Infection)	0/1	A PCR-confirmed infection with report of symptoms during the PCR referral / during the follow-up in the community setting / COVID-19 related hospitalization / COVID-19 related death.<br><br>Existing symptoms were considered when the physician or nurse checked the "symptomatic" option in the EMR, or when the following specific symptoms were recorded: fever or chills, cough, shortness of breath or difficulty breathing, sore throat, headache, weakness, congestion or runny nose, myalgia, nausea or vomiting, diarrhea, abdominal pain, loss of taste or smell,inability to eat or drink.	The date set for theSARS-CoV-2 infection outcome.
COVID-19 related	0/1		Date and time-of-

emergency room visit			day
COVID-19 related hospitalization	0/1	A hospitalization that was reported to the Israeli Ministry of Health (MOH) as a hospitalization of a SARS-CoV-2 infected individual.	The start date of the hospitalization.
COVID-19 related severe state	0/1	As defined by the hospitalizing institution per the Israeli MOH guidelines, consistent with the NIH criteria for severe illness or critical illness: Individuals who have oxygen saturation (SpO_2) <94% on room air at sea level, a ratio ofarterial partial pressure of oxygen to fraction of inspired oxygen (PaO_2/FiO_2) <300 mm Hg, respiratory frequency >30 breaths/min, or lung infiltrates >50%.<br><br>Critical Illness: Individuals who have respiratory failure, septic shock, and/or multiple organ dysfunction.	The first date during the hospitalization in which the individual was flagged as being in a severe or critical state.
COVID-19 related death	0/1	A death of a SARS-CoV-2 infected individual reported to the Israeli MOH.	The reported date of death.
COVID-19 vaccine-related side effect	0/1		
SARS-CoV-2-IgG serology post COVID-19 vaccine	0/1	Level of SARS-CoV-2-IgG	Date of the test and time-of-day

Supplemental Table 2: Clinical covariate definitions.

Variable	Values	Definitions	Timing
Age		Age in complete years	Current
Sex	Male/Female	As defined in files	Current
COVID Vaccine		Dose number, type	Date for each dose
COVID Vaccine			Time-of-day for each dose
Health-care worker	0/1	Is patient a health-care worker per File.	Current
Long-term care facility resident	0/1	Is patient a long-term care facility resident per file.	Current
Cancer	0/1	ICD9 Code 174*<br>ICD9 Code 175*<br>ICD9 Code 233.0<br>ICD9 Code V10.3<br>ICD9 Proc Code 85.4*<br>ICD9 Code 153*<br>ICD9 Code 154*<br>ICD9 Code V10.5*<br>ICD9 Code V10.6*<br>ICD9 Code 185<br>ICD9 Code V10.46<br>ICD9 Code 162*<br>ICD9 Code V10.1*<br>ICD9 Code 188*<br>ICD9 Code V10.51<br>ICD9 Code 183*<br>ICD9 Code V10.43<br>ICD9 Code 179<br>ICD9 Code 182*<br>ICD9 Code V10.42<br>ICD9 Code 157*<br>ICD9 Code 191*<br>ICD9 Code 192*<br>ICD9 Code V10.85<br>ICD9 Code 151*<br>ICD9 Code V10.04<br>ICD9 Code 172*<br>ICD9 Code V10.82<br>ICD9 Code 201*<br>ICD9 Code 200*<br>ICD9 Code 202.4*<br>ICD9 Code 204*<br>ICD9 Code 205*<br>ICD9 Code 206*<br>ICD9 Code 207.1*<br>ICD9 Code 208.1*	Last 5 years

		ICD9 Code 189*<br>ICD9 Code V10.52<br>ICD9 Code 160*<br>ICD9 Code 161*<br>ICD9 Code 164.0<br>ICD9 Code 195.0<br>ICD9 Code V10.21<br>ICD9 Code V10.22<br>ICD9 Code 180*<br>ICD9 Code V10.41<br>ICD9 Code 140*<br>ICD9 Code 141*<br>ICD9 Code 142*<br>ICD9 Code 143*<br>ICD9 Code 144*<br>ICD9 Code 145*<br>ICD9 Code 150*<br>ICD9 Code V10.03<br>ICD9 Code 155*<br>ICD9 Code 156*<br>ICD9 Code V10.07<br>ICD9 Code 170*<br>ICD9 Code V10.81<br>ICD9 Code 193<br>ICD9 Code V10.87<br>ICD9 Code 171*<br>ICD9 Code 176*<br>ICD9 Code 184*<br>ICD9 Code 186*<br>ICD9 Code 187*<br>ICD9 Code V10.4*<br>ICD9 Code 203*<br>ICD9 Code 273.3<br>ICD9 Code 152*<br>ICD9 Code 158*<br>ICD9 Code 159*<br>ICD9 Code 163*<br>ICD9 Code 164*<br>ICD9 Code 165*<br>ICD9 Code 181<br>ICD9 Code 190*<br>ICD9 Code 192.8<br>ICD9 Code 196*<br>ICD9 Code 197*<br>ICD9 Code 198*<br>ICD9 Code 199*	
Chronic Kidney Disease	0/1	ICD Proc Code 39.95<br>ICD Proc Code 54.98<br>ICD9 Code 996.81	Ever

		ICD9 Code V42.0<br>ICD Proc Code 55.6*<br>ICD9 Code 403._1<br>ICD9 Code 404._2<br>ICD9 Code 404._3<br>ICD9 Code 585*<br>ICD9 Code 586<br>ICD9 Code 250.4*<br>ICD9 Code 274.1*<br>ICD9 Code 440.1<br>ICD9 Code 581*<br>ICD9 Code 582*<br>ICD9 Code 583*<br>ICD9 Code 587<br>ICD9 Code 588*<br>ICD9 Code 589*	
Chronic Obstructive Pulmonary Disease	0/1	ICD9 Code 491*<br>ICD9 Code 492*<br>ICD9 Code 496	Ever
Heart Conditions	0/1	ICD9 Code 410*<br>ICD9 Code 411*<br>ICD9 Code 412<br>ICD9 Code 413*<br>ICD9 Code 414*<br>ICD9 Code 429.2, 429.7*<br>ICD9 Code V45.81, V45.82<br>ICD9 Proc Code 36.0*<br>ICD9 Proc Code 36.1*<br>ICD9 Code 428*<br>ICD9 Code 398.91<br>ICD9 Code 402._1<br>ICD9 Code 404._1,<br>ICD9 Code 404._3<br>ICD9 Code 416.9<br>ICD9 Code 514<br>ICD9 Code 425*<br>ICD9 Code 416*	Ever
Solid Organ Transplant Recipient	0/1	ICD9 Code 996.81<br>ICD9 Code V42.0<br>ICD Proc Code 55.6*<br>ICD9 Code V42.7<br>ICD Proc Code 50.5*<br>ICD9 Code V42.1<br>ICD9 Code V43.2<br>ICD Proc Code 37.5<br>ICD9 Code V42.83<br>ICD Proc Code52.8*<br>ICD9 Code V42.6	Ever

		ICD Proc Code33.5*<br>ICD Proc Code 33.6	
Obesity	0/1	Body Mass Index (BMI) 30-40	Latest measurement in last 5 years not taken during pregnancy
Severe Obesity	0/1	Body Mass Index (BMI) 40+	Latest measurement in last 5 years not taken during Pregnancy
Pregnancy	0/1		Current
Sickle Cell Disease	0/1	ICD9 Code 282.6*	Ever
Smoking	0/1		Last recorded value
Type 2<br>Diabetes<br>Mellitus	0/1	HbA1C > 6.5<br>ATC Codes A10[A,B]<br>ICD9 Code 250*<br>ICD9 Code 357.2<br>ICD9 Code 362.0*<br><br>**And not:**<br>ICD9 Code 250._1, 250._3	For diagnosis codes, ever<br><br>For drugs, 4 or more dispensed in last 12 months
Asthma	0/1	ICD9 Code 493*	Ever
Cerebrovascular Disease	0/1	ICD9 Code 362.34<br>ICD9 Code 430<br>ICD9 Code 431<br>ICD9 Code 432*<br>ICD9 Code 433*<br>ICD9 Code 434*<br>ICD9 Code 435*<br>ICD9 Code 436*<br>ICD9 Code 438*	Ever
Other Respiratory Disease	0/1	ICD9 Code 277.0*<br>ICD9 Code 494*<br>ICD9 Code 515	Ever
Hypertension	0/1	ICD9 Code 401*<br>ICD9 Code 402*<br>ICD9 Code 403*<br>ICD9 Code 404*<br>ICD9 Code 405*	Ever
Immunocompromised State	0/1	**Any of:**<br>ICD9 Code 042*<br>ICD9 Code 043*<br>ICD9 Code 044*<br>ICD9 Code<br>795.71 ICD9<br>Code V08 ICD9<br>Code V42.8*<br>ICD9 Proc Code 41.0*<br><br>**Or at least 2 of:**	For diagnosis codes, Ever<br>For drugs, 4 or more dispensed in last 12 months

		ATC4 Code<br>H02ABATC4 Code<br>H02BXATC4 Code<br>M01BA<br><br>**Or at least 2 of:**<br>ATC2 Code L04	

Supplemental Table 3: Results of Schoenfeld's Global test for proportional hazards assumption

	Model includes all ages			Model includes individuals ≥60 years of age		
**Variable**	**Chisq**	**df**	**P**	**Chisq**	**df**	**p-Value**
Sex	0.36	1	0.55	0.0000455	1	0.99
Age	1804.67	1	<0.001	-	-	-
Morning vs Evening	138.27	1	<0.001	10.2	1	0.001
Diabetes	515.29	1	<0.001	2.46	1	0.12
Obesity	396.19	1	<0.001	5.77	1	0.02
Chronic kidney disease	212.85	1	<0.001	0.87	1	0.35
Hypertension	1478.49	1	<0.001	0.98	1	0.32
Global test	2608.96	7	<0.001	17.3	6	0.01

Supplemental Table 4: Multivariate logistic regression model for association with COVID breakthrough infection post vaccine doses 1 and 2.

Variable	Odds Ratio	Confidence Interval (5%-95%)	p-Value
Third vaccine (booster)	0.80	0.79-0.82	<0.001
Male gender	0.81	0.80-0.82	<0.001
Evening doses	1.02	1.01-1.04	0.006
Diabetes Mellitus	0.97	0.93-1.00	0.07
Age > 60y	0.68	0.66-0.70	<0.001
Obesity	1.00	0.98-1.02	0.6
Chronic Kidney Disease	0.90	0.85-0.95	<0.001
Hypertension	1.02	0.99-1.05	0.14

Supplemental Table 5: Bootstrap multivariate analysis (2000 rounds) for the association with COVID infection post two vaccine doses.

Variable	Odds Ratio	Confidence Interval (5%-95%)
Third vaccine (booster)	0.28	0.27-0.30
Male gender	0.82	0.80-0.83
Evening doses	1.07	1.06-1.09
Diabetes Mellitus	0.96	0.92-0.99
Age > 60y	0.52	0.50-0.55
Obesity	0.91	0.90-0.93
Chronic Kidney Disease	0.90	0.86-0.95
Hypertension	0.95	0.92-0.97

Supplemental Table 6: Cox regression analysis of breakthrough infection after the initial vaccine series given different permutations of dosing with time of day (n=1,515,754).

	Doses 1 and 2		
	HR	95% CI	p
**Booster Dose[a]**			
**-**	—	—	
**+**	0.25	0.24-0.25	<0.001
**Vaccination Timing**			
**AM/AM**	—	—	—
**PM/AM**	0.93	0.91-0.95	<0.001
**AM/PM**	1.08	1.05-1.11	<0.001
**PM/PM**	1.04	1.02-1.06	<0.001
**Sex**			
**F**	—	—	
**M**	0.83	0.82-0.84	<0.001
**Age[e]**			
**≤60**	—	—	
**>60**	0.19	0.0.18-0.19	<0.001
**Diabetes**			
**-**	—	—	
**+**	0.72	0.71-0.74	<0.001
**BMI[b,e]**			
**<30**	—	—	
**≥30**	0.87	0.86-0.88	<0.001
**CKD[c]**			
**-**	—	—	
**+**	0.86	0.84-0.89	<0.001
**HTN[d]**			
**-**	—	—	
**+**	0.72	0.71-0.74	<0.001

[a]Models the effect one additional booster dose beyond the number of vaccinations specified.

[b]BMI, body mass index; [c]CKD, chronic kidney disease; [d]HTN, hypertension. [e]Modeling BMI and age as continuous variables yielded similar trends (data not shown).

Supplemental Table 7: Cox regression with mixed random effects analysis of <u>breakthrough infection</u> after COVID-19 vaccination: morning vs. evening.

	Dose 2			Dose 3			Dose 4		
	HR	95% CI	p	HR	95% CI	p	HR	95% CI	p
**Booster Dose**[a]									
-	—	—		—	—		—	—	
+	0.25	0.24-0.25	<0.001	0.44	0.42-0.45	<0.001	—	—	
**Vaccination Timing**									
8:00-11:59	0.95	0.94-0.96	<0.001	0.97	0.95-0.98	<0.001	0.97	0.93-1.02	0.3
12:00-15:59	0.92	0.91-0.93	<0.001	0.93	0.91-0.94	<0.001	0.93	0.88-0.97	0.003
16:00-19:59	—	—		—	—		—	—	—
**Sex**									
F	—	—		—	—		—	—	
M	0.83	0.83-0.84	<0.001	0.83	0.82-0.84	<0.001	1.13	1.08-1.19	<0.001
**Age**[e]									
≤60	—	—		—	—		—	—	
>60	0.19	0.0.18-0.19	<0.001	0.68	0.67-0.70	<0.001	1.17	1.10-1.24	<0.001
**Diabetes**									
-	—	—		—	—		—	—	
+	0.72	0.71-0.74	<0.001	0.91	0.89-0.94	<0.001	0.89	0.84-0.94	<0.001
**BMI**[b,e]									
<30	—	—		—	—		—	—	
≥30	0.87	0.86-0.88	<0.001	0.94	0.93-0.95	<0.001	0.95	0.90-1.00	0.052
**CKD**[c]									
-	—	—		—	—		—	—	
+	0.86	0.84-0.89	<0.001	0.91	0.88-0.94	<0.001	0.84	0.78-0.89	<0.001
**HTN**[d]									
-	—	—		—	—		—	—	
+	0.72	0.71-0.74	<0.001	0.93	0.91-0.95	<0.001	0.93	0.88-0.98	0.004

[a]Models the effect one additional booster dose beyond the number of vaccinations specified.

[b]BMI, body mass index; [c]CKD, chronic kidney disease; [d]HTN, hypertension. [e]Modeling BMI and age as continuous variables yielded similar trends (data not shown).

Supplemental Table 8: Cox regression with mixed random effects analysis of COVID-19 associated emergency department (ED) visits after vaccination.

	Dose 2			Dose 3			Dose 4		
	HR	95% CI	p	HR	95% CI	p	HR	95% CI	p
**Booster Dose[b]**									
**-**	—	—		—	—		—	—	
**+**	0.20	0.18-0.22	<0.001	0.33	0.27-0.41	<0.001	—	—	
**Vaccination Timing**									
**8:00-11:59**	0.92	0.83-1.03	0.2	0.94	0.80-1.10	0.4	0.69	0.45-1.04	0.076
**12:00-15:59**	0.98	0.87-1.11	0.8	1.02	0.87-1.20	0.8	0.96	0.58-1.31	0.5
**16:00-19:59**	—	—		—	—	—	—	—	
**Age[f]**									
**≤60**	—	—		—	—		—	—	
**>60**	1.66	1.41-1.95	<0.001	1.99	1.64-2.42	<0.001	1.49	0.70-3.16	0.3
**BMI[c,f]**									
**≥30**	—	—		—	—		—	—	
**<30**	1.23	1.08-1.40	0.002	1.01	0.86-1.20	0.9	0.7	0.50-1.19	0.2
**Diabetes**									
**-**	—	—		—	—		—	—	
**+**	1.55	1.31-1.84	<0.001	1.51	1.24-1.85	<0.001	2.09	1.38-3.16	<0.001
**HTN[d]**									
**-**	—	—		—	—		—	—	
**+**	1.32	1.12-1.56	0.046	1.36	1.11-1.65	0.002	1.64	1.00-2.69	0.051
**CKD[e]**									
**-**	—	—		—	—		—	—	
**+**	2.35	1.96-2.83	<0.001	2.63	2.11-3.27	<0.001	2.54	1.62-3.96	<0.001
**Immuno-suppression**									
**-**	—	—		—	—		—	—	
**+**	4.65	3.92-5.52	<0.001	5.21	4.25-6.40	<0.001	5.29	3.44-8.14	<0.001

[a]Models the effect one additional booster dose beyond the number of vaccinations specified.

[b]BMI, body mass index; [c]CKD, chronic kidney disease; [d]HTN, hypertension. [e]Modeling BMI and age as continuous variables yielded similar trends (data not shown).

Supplemental Table 9: Cox regression with mixed random effects analysis of COVID-19 associated hospitalizations after vaccination.

	Dose 2			Dose 3			Dose 4		
	HR	95% CI	p	HR	95% CI	p	HR	95% CI	p
**Booster Dose**[b]									
**-**	—	—		—	—		—	—	
**+**	0.19	0.17-0.21	<0.001	0.36	0.30-0.43	<0.001	—	—	
**Vaccination Timing**									
**8:00-11:59**	0.90	0.78-1.04	0.15	0.93	0.74-1.16	0.5	0.64	0.43-0.97	0.038
**12:00-15:59**	0.99	0.86-1.14	0.9	0.96	0.78-1.20	0.7	0.87	0.58-1.31	0.5
**16:00-19:59**	—	—		—	—	—	—	—	
**Age**[f]									
**≤60**	—	—		—	—		—	—	
**>60**	2.06	1.76-2.40	<0.001	2.19	1.18-2.65	<0.001	1.08	0.59-0.97	0.8
**BMI**[c,f]									
**≥30**	—	—		—	—		—	—	
**<30**	1.26	1.12-1.42	<0.001	0.97	0.82-1.14	0.7	0.63	0.42-0.92	0.018
**Diabetes**									
**-**	—	—		—	—		—	—	
**+**	1.57	1.35-1.82	<0.001	1.40	1.16-1.69	<0.001	1.78	1.25-2.53	0.001
**HTN**[d]									
**-**	—	—		—	—		—	—	
**+**	1.43	1.22-1.67	0.046	1.38	1.15-1.66	<0.001	1.95	1.25-3.04	0.003
**CKD**[e]									
**-**	—	—		—	—		—	—	
**+**	2.48	2.11-2.93	<0.001	2.76	2.25-3.38	<0.001	3.00	2.03-4.42	<0.001
**Immuno-suppression**									
**-**	—	—		—	—		—	—	
**+**	5.21	4.48-6.07	<0.001	6.36	5.29-7.66	<0.001	5.63	3.91-8.11	<0.001

[a]Models the effect one additional booster dose beyond the number of vaccinations specified.

[b]BMI, body mass index; [c]CKD, chronic kidney disease; [d]HTN, hypertension. [e]Modeling BMI and age as continuous variables yielded similar trends (data not shown).

Supplemental Table 10: Patient demographic breakdown for periodicity analysis of breakthrough infections (Fig. 3)

	Doses 1 and 2			Dose 3			Dose 4		
**Time Bin**	**Mean Age (SD)**	**Male (%)**	**≥ 4 Comorbidities**	**Mean Age (SD)**	**Male (%)**	**≥ 4 Comorbidities**	**Mean Age (SD)**	**Male (%)**	**≥ 4 Comorbidities**
**8:00-9:59**	45.8 (18.7)	48.8%	1.74%	47.3 (17.8)	47.9%	1.8%	68.3 (11.2)	52.5%	5.7%
**10:00-11:59**	45.3 (19.9)	47.7%	2.0%	46.4 (18.6)	46.9%	1.9%	69.8 (11.1)	49.1%	6.4%
**11:00-12:59**	45.1 (20.0)	47.7%	1.96%	46.3 (18.6)	47.4%	1.8%	70.3 (10.8)	48.6%	6.4%
**12:00-13:59**	44.7 (19.9)	47.4%	1.87%	47.0 (18.7)	47.8%	1.9%	70.6 (10.4)	48.6%	6.4%
**13:00-14:59**	43.4 (20.0)	47.1%	1.69%	47.2 (18.9)	48.1%	1.9%	69.7 (11.2)	48.7%	6.2%
**14:00-15:59**	42.3 (20.1)	47.0%	1.55%	46.2 (19.2)	47.8%	1.9%	68.5 (11.9)	49.7%	5.9%
**15:00-16:59**	41.0 (20.0)	46.8%	1.41%	44.7 (19.4)	47.0%	1.7%	67.1 (12.3)	50.6%	5.6%
**16:00-17:59**	40.0 (19.7)	47.4%	1.23%	43.8 (19.3)	47.6%	1.5%	65.6 (12.6)	51.9%	5.2%
**17:00-18:59**	40.3 (19.1)	48.8%	1.14%	43.5 (19.0)	49.1%	1.4%	65.0 (12.3)	54.4%	4.8%
**18:00-19:59**	41.2 (18.3)	50.0%	1.12%	43.9 (18.7)	50.3%	1.4%	65.7 (11.4)	55.7%	4.8%
**19:00-20:59**	41.4 (17.6)	50.1%	1.04%	45.0 (18.1)	50.8%	1.3%	67.3 (10.2)	55.3%	5.3%
**20:00-20:59**	40.1 (17.0)	49.6%	0.82%	45.5 (17.4)	51.9%	1.3%	68.4 (9.1)	55.6%	6.0%

Supplemental Table 11: Breakthrough COVID-19 infection hazard ratio (HR) comparing AM (8:00-9:59) vaccination to the indicated times. Ratios <1 indicate fewer infections relative to the reference time bin (8:00-9:59). For graphical representation of these data see Figure 3A-C.

Time Bin	Dose 2 HR	Dose 2 95% CI	Dose 2 n	Dose 3 HR	Dose 3 95% CI	Dose 3 n	Dose 4 HR	Dose 4 95% CI	Dose 4 n
08:00-09:59	1.00		98788	1.00		186932	1.00		29870
10:00-11:59	0.93	0.91-0.95	258861	0.98	0.96-0.99	204203	0.99	0.92-1.05	35345
11:00-12:59	0.92	0.90-0.94	234150	0.97	0.96-0.99	191679	0.96	0.89-1.02	32442
12:00-13:59	0.91	0.88-0.93	211139	0.94	0.93-0.96	181319	0.96	0.89-1.02	32471
13:00-14:59	0.94	0.92-0.96	202241	0.93	0.92-0.95	173612	0.90	0.84-0.96	36958
14:00-15:59	0.96	0.93-0.98	207377	0.96	0.95-0.98	170538	0.90	0.84-0.96	42222
15:00-16:59	1.01	0.98-1.03	218308	1.00	0.98-1.01	186127	0.94	0.89-1.01	47780
16:00-17:59	1.07	1.04-1.09	226135	1.02	1.00-1.03	206931	0.99	0.93-1.06	47576
17:00-18:59	1.06	1.03-1.08	202588	1.02	1.01-1.04	179406	1.04	0.98-1.11	35991
18:00-19:59	0.99	0.97-1.01	148651	1.03	1.01-1.05	114029	1.08	1.00-1.17	18538
19:00-20:59	0.96	0.93-0.99	72019	1.02	0.99-1.04	54557	1.16	1.05-1.29	6138
20:00-21:59	0.87	0.82-0.93	16900	1.01	0.97-1.05	16326	1.16	0.95-1.42	1458

Supplemental Table 12: COVID-19 associated ED visit hazard ratio (HR) comparing AM (8:00-9:59) vaccination to the indicated times. Ratios <1 indicate fewer infections relative to the reference time bin (8:00-9:59). For graphical representation of these data see Supplemental Figure 6.

Time Bin	Dose 2 HR	Dose 2 95% CI	Dose 2 n	Dose 3 HR	Dose 3 95% CI	Dose 3 n	Dose 4 HR	Dose 4 95% CI	Dose 4 n
08:00-09:59	1.00		98788	1.00		186932			29870
10:00-11:59	0.87	0.74-1.02	258861	0.88	0.73-1.05	204203	1.07	0.73 - 1.56	35345
11:00-12:59	0.93	0.79-1.10	234150	0.90	0.75-1.08	191679	1.45	1.01 - 2.07	32442
12:00-13:59	1.02	0.87-1.21	211139	0.88	0.73-1.07	181319	1.44	0.99 - 2.1	32471
13:00-14:59	0.97	0.82-1.15	202241	0.82	0.68-1.00	173612	1.13	0.75 - 1.72	36958
14:00-15:59	0.94	0.79-1.12	207377	0.79	0.64-0.96	170538	0.82	0.51 - 1.32	42222
15:00-16:59	0.93	0.78-1.11	218308	0.95	0.79-1.15	186127	0.79	0.49 - 1.28	47780
16:00-17:59	1.00	0.85-1.19	226135	0.89	0.74-1.07	206931	0.81	0.49 - 1.32	47576
17:00-18:59	0.97	0.81-1.16	202588	0.80	0.66-0.98	179406	0.80	0.48 - 1.34	35991
18:00-19:59	0.91	0.75-1.11	148651	0.91	0.73-1.14	114029	1.07	0.63 - 1.8	18538
19:00-20:59	1.02	0.50-1.45	72019	1.10	0.84-1.44	54557	1.04	0.51 - 2.14	6138
20:00-21:59	0.85	0.17-1.23	16900	1.28	0.84-1.95	16326	1.83	0.56 - 5.98	1458

Supplemental Table 13: COVID-19 associated hospitalization hazard ratio (HR) comparing AM (8:00-9:59) vaccination to the indicated times. Ratios <1 indicate fewer infections relative to the reference time bin (8:00-9:59). For graphical representation of these data see Figure 3D and Supplemental Figure 6.

Time Bin	Dose 2 HR	Dose 2 95% CI	Dose 2 n	Dose 3 HR	Dose 3 95% CI	Dose 3 n	Dose 4 HR	Dose 4 95% CI	Dose 4 n
08:00-09:59	1.00		98788	1.00		186932	1.00		29870
10:00-11:59	0.91	0.76 - 1.1	258861	0.92	0.77 - 1.09	204203	1.07	0.73 - 1.57	35345
11:00-12:59	0.98	0.82 - 1.18	234150	1.04	0.88 - 1.24	191679	1.38	0.95 - 2	32442
12:00-13:59	1.10	0.91 - 1.32	211139	1.17	0.98 - 1.4	181319	1.39	0.94 - 2.04	32471
13:00-14:59	1.03	0.85 - 1.25	202241	1.06	0.88 - 1.28	173612	1.08	0.7 - 1.66	36958
14:00-15:59	0.96	0.79 - 1.17	207377	0.92	0.75 - 1.12	170538	0.72	0.44 - 1.2	42222
15:00-16:59	0.91	0.75 - 1.12	218308	0.85	0.7 - 1.05	186127	0.72	0.43 - 1.19	47780
16:00-17:59	1.01	0.83 - 1.23	226135	0.96	0.79 - 1.17	206931	0.77	0.46 - 1.28	47576
17:00-18:59	1.00	0.82 - 1.23	202588	0.96	0.79 - 1.18	179406	0.79	0.47 - 1.34	35991
18:00-19:59	0.88	0.7 - 1.11	148651	0.88	0.69 - 1.11	114029	1.11	0.66 - 1.88	18538
19:00-20:59	0.93	0.69 - 1.26	72019	0.84	0.61 - 1.15	54557	1.92	0.53 - 2.25	6138
20:00-21:59	0.94	0.51 - 1.72	16900	0.97	0.53 - 1.78	16326	3.35	0.57 - 6.29	1458

Supplemental Table 14: METACYLE output for data presented in Figures 3, 4, and Supplemental Figure 6.

COVID Vaccination Number	Age Range	Outcome	meta2d pvalue	meta2d BH.Q	meta2d period	meta2d phase	meta2d Base	meta2d AMP	meta2d rAMP
**Dose 2**	All	Infection	0.002	0.004	7.433	0.015	0.999	0.057	0.057
	12-30 Years	Infection	0.003	0.004	7.199	7.002	0.998	0.100	0.100
	30-60 Years	Infection	0.001	0.003	7.130	0.812	0.907	0.044	0.044
	>60 Years	Infection	0.034	0.034	6.021	4.940	1.002	0.054	0.054
	All	ED Visit	0.153	0.153	11.466	5.402	0.928	0.105	0.105
	All	Hospitalization	0.716	0.894	11.511	7.736	0.969	0.011	0.011
**Dose 3**	All	Infection	0.003	0.006	14.823	11.964	0.993	0.017	0.017
	12-30 Years	Infection	<0.001	0.003	15.157	12.184	1.009	0.006	0.006
	30-60 Years	Infection	0.020	0.028	13.554	13.532	0.966	0.011	0.011
	>60 Years	Infection	0.006	0.010	16.747	13.022	1.048	0.062	0.060
	All	ED Visit	0.152	0.153	14.055	10.113	0.928	0.022	0.022
	All	Hospitalization	0.894	0.894	11.512	8.242	0.962	0.098	0.098
**Dose 4**	All	Infection	<0.001	<0.001	16.077	13.563	1.049	0.105	0.100
	All	ED Visit	0.004	0.016	14.950	4.596	1.542	0.904	0.586
	All	Hospitalization	0.009	0.018	13.969	4.069	1.159	0.278	0.240

Supplemental Table 15: Breakthrough COVID-19 infection hazard ratio (HR) as a function of age bracket, comparing AM (8:00-11:59) to PM (16:00-19:59) vaccination times. Ratios <1 indicate fewer infections associated with the AM vaccination. For graphical representation of these data see Figure 4A.

	Dose 2			Dose 3		
**Age Bin [Years]**	**HR**	**95% CI**	**Total n**	**HR**	**95% CI**	**Total n**
**12-20**	0.90	0.88-0.93	139985	0.95	0.92-0.99	81773
**20-30**	0.99	0.97-1.02	130322	0.98	0.95-1.01	100108
**30-40**	0.99	0.96-1.01	141686	0.99	0.96-1.01	112924
**40-50**	0.98	0.96-1.01	162474	0.98	0.95-1.01	138923
**50-60**	0.92	0.90-0.95	145675	0.93	0.91-0.99	129752
**>60**	0.92	0.90-0.95	207304	0.95	0.92-0.98	192730

Supplemental Table 16: Breakthrough COVID-19 infection hazard ratio (HR) as a function of age bracket, comparing afternoon (12:00-15:59) to PM (16:00-19:59) vaccination times. Ratios <1 indicate fewer infections associated with the afternoon vaccination. For graphical representation of these data see Supplemental Figure 7.

	Dose 2			Dose 3		
**Age Bin [Years]**	**HR**	**95% CI**	**Total n**	**HR**	**95% CI**	**Total n**
**12-20**	0.87	0.84-0.89	137659	0.91	0.88-0.94	80100
**20-30**	0.94	0.92-0.97	116663	0.95	0.92-0.98	88219
**30-40**	0.94	0.92-0.97	108794	0.95	0.92-0.98	85352
**40-50**	0.95	0.93-0.98	119668	0.97	0.95-1.01	100930
**50-60**	0.93	0.91-0.96	108443	0.95	0.92-0.98	95909
**>60**	0.95	0.92-0.99	134157	0.97	0.93-1.01	124829

Supplemental Table 17: Breakthrough COVID-19 infection hazard ratio (HR) in patients 12-30 years old, comparing AM (8:00-9:59) vaccination to the indicated times. Ratios <1 indicate fewer infections relative to the reference time bin (8:00-9:59). For graphical representation of these data see Figure 4B.

	Dose 2			Dose 3		
**Time Bin**	**HR**	**95% CI**	**n**	**HR**	**95% CI**	**n**
**08:00-09:59**	1.00		25502	1.00		35445
**10:00-11:59**	0.94	0.90-0.97	27084	0.97	0.93-1.00	47677
**11:00-12:59**	0.90	0.87-0.94	25632	0.96	0.93-0.99	45992
**12:00-13:59**	0.88	0.85-0.92	22992	0.92	0.89-0.96	41710
**13:00-14:59**	0.90	0.86-0.93	24923	0.92	0.89-0.96	39803
**14:00-15:59**	0.90	0.86-0.94	29103	0.98	0.95-1.01	42315
**15:00-16:59**	0.98	0.94-1.02	34537	1.04	1.00-1.07	51461
**16:00-17:59**	1.08	1.05-1.13	39777	1.07	1.04-1.11	59998
**17:00-18:59**	1.05	1.01-1.09	34995	1.09	1.05-1.13	52446
**18:00-19:59**	0.94	0.91-0.98	23416	1.10	1.06-1.14	31950
**19:00-20:59**	0.88	0.83-0.92	10075	1.06	1.01-1.11	13738
**20:00-21:59**	0.80	0.73-0.89	2107	1.00	0.93-1.09	3810

Supplemental Table 18: Breakthrough COVID-19 infection hazard ratio (HR) in patients 30-60 years old, comparing AM (8:00-9:59) vaccination to the indicated times. Ratios <1 indicate fewer infections relative to the reference time bin (8:00-9:59). For graphical representation of these data see Figure 4C.

	Dose 2			Dose 3		
**Time Bin**	**HR**	**95% CI**	**n**	**HR**	**95% CI**	**n**
**08:00-09:59**	1.00		58496	1.00		103783
**10:00-11:59**	0.93	0.91-0.96	42817	0.99	0.97-1.01	105491
**11:00-12:59**	0.92	0.90-0.95	38015	0.97	0.95-0.99	98468
**12:00-13:59**	0.91	0.88-0.94	33272	0.95	0.93-0.97	92407
**13:00-14:59**	0.94	0.91-0.97	31863	0.93	0.91-0.95	86538
**14:00-15:59**	0.95	0.93-0.98	33655	0.95	0.93-0.97	83818
**15:00-16:59**	0.98	0.95-1.01	37148	0.96	0.94-0.98	90551
**16:00-17:59**	1.00	0.97-1.03	42052	0.97	0.95-0.99	101528
**17:00-18:59**	1.01	0.98-1.04	41927	0.97	0.95-0.99	88891
**18:00-19:59**	0.99	0.96-1.02	34695	0.98	0.95-1.00	57329
**19:00-20:59**	1.00	0.96-1.04	17022	0.97	0.94-1.00	28452
**20:00-21:59**	0.91	0.84-0.98	3346	0.97	0.92-1.02	8778

Supplemental Table 19: Breakthrough COVID-19 infection hazard ratio (HR) in patients >60 years old, comparing AM (8:00-9:59) vaccination to the indicated times. Ratios <1 indicate fewer infections relative to the reference time bin (8:00-9:59). For graphical representation of these data see Figure 4D.

	Dose 2			Dose 3		
**Time Bin**	**HR**	**95% CI**	**n**	**HR**	**95% CI**	**n**
**08:00-09:59**	1.00		14789	1.00		47704
**10:00-11:59**	0.84	0.78-0.90	13622	0.97	0.93-1.01	51035
**11:00-12:59**	0.89	0.83-0.96	11593	0.99	0.95-1.03	47219
**12:00-13:59**	0.91	0.85-0.98	9662	0.96	0.92-1.00	47202
**13:00-14:59**	0.94	0.86-1.01	8488	0.94	0.90-0.98	47271
**14:00-15:59**	1.00	0.92-1.08	8354	0.97	0.93-1.01	44405
**15:00-16:59**	0.95	0.88-1.03	8658	1.01	0.97-1.05	44115
**16:00-17:59**	0.97	0.90-1.05	8847	1.04	1.00-1.09	45405
**17:00-18:59**	1.07	0.99-1.16	8101	1.05	1.01-1.10	38069
**18:00-19:59**	1.07	0.99-1.16	6214	1.06	1.01-1.12	24750
**19:00-20:59**	1.03	0.92-1.16	2672	1.09	1.02-1.16	12367
**20:00-21:59**	0.98	0.75-1.28	434	1.19	1.08-1.32	3738

Supplemental Table 20: Distribution of COVID-19 tests in the study population. See Supplemental Figure 3 for a graphical presentation of these data.

Hour Bin	Total Tests		Positive Tests		Positivity Rate	
	PM[a]	AM[a]	PM	AM	PM	AM
0	18301	27141	509	779	0.028	0.029
1	6218	8880	52	92	0.008	0.010
2	5496	7990	33	52	0.006	0.007
3	6212	8998	48	63	0.008	0.007
4	3776	5464	50	75	0.013	0.014
5	1951	2553	42	58	0.022	0.023
6	9333	13285	55	101	0.006	0.008
7	7562	11183	255	337	0.034	0.030
8	23481	35000	1970	2552	0.084	0.073
9	36674	55411	3518	4766	0.096	0.086
10	41152	61818	4267	5804	0.104	0.094
11	42229	60857	4240	5895	0.100	0.097
12	46097	64411	4879	6246	0.106	0.097
13	39846	52965	3982	5017	0.100	0.095
14	35845	45797	3448	4197	0.096	0.092
15	34279	42387	2993	3469	0.087	0.082
16	35709	40957	2903	3145	0.081	0.077
17	38150	41949	2894	2900	0.076	0.069
18	32168	34999	2632	2691	0.082	0.077
19	25905	28451	2260	2274	0.087	0.080
20	19160	23005	1405	1488	0.073	0.065
21	15003	18950	945	1036	0.063	0.055
22	9372	12952	291	322	0.031	0.025
23	8142	11692	99	136	0.012	0.012

[a]AM:8:00-11:59; PM:16:00-19:59
