# Supplementary material for: Biological rhythms in COVID-19 vaccine effectiveness in an observational cohort study of 1.5 million patients
Source: J Clin Invest. 2023 Jun 1;133(11):e167339. doi: 10.1172/JCI167339 (PMC10231992; doi:10.1172/JCI167339)
Supplement: Trial reporting checklists [file jci-133-167339-s154.pdf]

STROBE REPORTING CHECKLIST FOR OBSERVATIONAL STUDIES

Strobe Checklist Item	Manuscript
**Title and Abstract**	
(a) Indicate the study's design with a commonly used term in the title or the abstract<br>(b) Provide in the abstract an informative and balanced summary of what was done and what was found	Title "Biological rhythms in COVID-19 vaccine effectiveness in an observational cohort study of 1.5 million patients."<br><br>Abstract consistent with STROBE guidelines.
**Results- Participants**	
(a) Report numbers of individuals at each stage of study—eg numbers potentially eligible, examined for eligibility, confirmed eligible, included in the study, completing follow-up, and analysed.<br>(b) Give reasons for non-participation at each stage<br>(c) Consider use of a flow diagram.	**Fig 1A** provides a flow diagram of this information.
**Results- Descriptive Data**	
(a) Give characteristics of study participants (eg demographic, clinical, social) and information on exposures and potential confounders<br>(b) Indicate number of participants with missing data for each variable of interest<br>(c) Summarise follow-up time (eg, average and total amount)	**Table 1** provides this information. There is no missing data for the variables of interest.
**Results- Outcome Data**	
Report numbers of outcome events or summary measures over time.	These data are contained in the main text, **Fig. 2**, and **Supplemental Figs. 1-4**.
**Results- Main Results**	
(a) Give unadjusted estimates and, if applicable, confounder-adjusted estimates and their precision (eg, 95% confidence interval). Make clear which confounders were adjusted for and why they were included.<br>(b) Report category boundaries when continuous variables were categorized<br>(c) If relevant, consider translating estimates of relative risk into absolute risk for a meaningful time period.	These data can be found in the main text, **Figs. 1-4**, and **Supplemental Tables 7-9**.

**Results- Other Analyses**	
Report other analyses done—eg analyses of subgroups and interactions, and sensitivity analyses	These can be found in **Figs. 3, 4, and Supplemental Figures 6 and 7; Supplemental Tables 3-5, and 11-19**.
**Discussion- Key Results**	
Summarise key results with reference to study objectives	Included in the Discussion section.
**Discussion- Limitations**	
Discuss limitations of the study, taking into account sources of potential bias or imprecision. Discuss both direction and magnitude of any potential bias	Included in the Discussion section.
**Discussion- Interpretation**	
Give a cautious overall interpretation of results considering objectives, limitations, multiplicity of analyses, results from similar studies, and other relevant evidence	Included in the Discussion section.
**Discussion- Generalizability**	
Discuss the generalisability (external validity) of the study results	Included in the Discussion section.
**Other Information**	
Give the source of funding and the role of the funders for the present study and, if applicable, for the original study on which the present article is based	Included in the Funding section at the end of the document.
