# Supplementary material for: Biological rhythms in COVID-19 vaccine effectiveness in an observational cohort study of 1.5 million patients
Source: J Clin Invest. 2023 Jun 1;133(11):e167339. doi: 10.1172/JCI167339 (PMC10231992; doi:10.1172/JCI167339)
Supplement: Supplemental data set 1 [file jci-133-167339-s156.pdf]

Supplemental Methods: R code used for data analysis.

BIOLOGICAL RHYTHMS IN COVID-19 VACCINE EFFECTIVENESS, AN OBSERVATIONAL COHORT STUDY OF 1.5 MILLION PATIENTS

Guy Hazan, M.D., Ph.D.[1-3], Or A. Duek, PhD[4,5], Hillel Alapi, B.A.[6], Huram Mok, MD[1], Alex Ganninger[1], Elaine Ostendorf[1], Carrie Gierasch, B.S.[1], Gabriel Chodick, Ph.D.[6,7], David Greenberg, M.D.[8,9], and Jeffrey A. Haspel, M.D., Ph.D.[1*].

[1]Division of Pulmonary and Critical Care Medicine, Department of Internal Medicine, Washington University School of Medicine, St. Louis, MO, USA.

[2]Department of Pediatrics, Soroka University Medical Center, Beer-Sheva, Israel.

[3]Research and Innovation Center, Saban Childrens Hospital, Beer-Sheva, Israel.

[4]Department of Psychiatry, Yale University School of Medicine, New Haven, CT, USA.

[5]Department of Epidemiology, Biostatistics and Community Health Sciences, Faculty of Health Sciences, Ben-Gurion University of the Negev, Beer-Sheva, Israel

[6]Maccabitech Institute for Research and Innovation, Maccabi Healthcare Services, Tel Aviv, Israel.

[7]Sackler Faculty of Medicine, Tel Aviv University, Tel Aviv, Israel.

[8]The Pediatric Infectious Disease Unit, Soroka University Medical Center, Beer-Sheva, Israel.

[9]Faculty of Health Sciences, Ben-Gurion University of the Negev, Beer-Sheva, Israel.

*Corresponding author contact information: Jeffrey Haspel, Division of Pulmonary and Critical Care Medicine, Department of Internal Medicine, Washington University School of Medicine, Campus Box 8052, 660 South Euclid Avenue, St. Louis, MO, 63110, USA. Email: jhaspel@wustl.edu

```
####Data Cleaning####

install.packages("tidyverse")
library("tidyverse")

install.packages("chron")
library (chron)

install.packages("lubridate")
library("lubridate")

install.packages("eeptools")
library("eeptools")

install.packages("pastecs")
library ("pastecs")

install.packages("epiDisplay")
library("epiDisplay")

install.packages("dplyr")
library("dplyr")

install.packages("tidyr")
library("tidyr")

library ("ggplot2")
###Read files####

Diagnostic_test <- read.table ("Circadian_Clock_All_PCR.csv",sep = "\t",
header = TRUE)
Population <- read.table
("Circadian_Clock_Covid19_Population_Final.csv",sep = "\t", header =
TRUE)
Hospitalization <- read.table
("Circadian_Clock_Corona_Hosp_Final.csv",sep = "\t", header = TRUE)
Hospital_diagnosis <- read.table
("Circadian_Clock_CORONA_HOSPITAL_DIAGNOSIS.csv",sep = "\t", header =
TRUE)
Symptoms <- read.table ("Circadian_Clock_Covid_Symp_Final.csv",sep =
"\t", header = TRUE)
ER <- read.table ("Circadian_Clock_Covid19_ER.csv",sep = "\t", header =
TRUE)
COVID_Variant <- read.table
("Circadian_Clock_Covid19_Variant_Final.csv",sep = "\t", header = TRUE)
Home_Hospitalization <- read.table
("Circadian_Clock_Home_Hosp_Final.csv",sep = "\t", header = TRUE)
PCR_Result <- read.table ("Circadian_Clock_PCR_Gene_Final.csv",sep =
"\t", header = TRUE)
Side_Effect <- read.table
("Circadian_Clock_Vaccine_Side_effect_Final.csv",sep = "\t", header =
TRUE)

#Data cleaning was done and final files were created :
```

```
###general.csv - file with exclusion of patients that were infected prior
to dose 1 + 7 days
###general2.csv - file with exclusion of patients that were infected
prior to dose 2+ 7 days
###general3.csv - file with exclusion of patients that were infected
prior to dose 3 + 7 days
###general4.csv - file with exclusion of patients that were infected
prior to dose 4 + 7 days

#####Descriptive analysis######
## loading data
require(tidyverse)
require(gtsummary)
require(table1)

# call data organizing function
source('organizeData.r')

df2 <- read.csv('general2.csv')

df2$hourBin <- case_when((df2$Hour2>=8 & df2$Hour2<=11) ~ 'Morning',
                         (df2$Hour2>=16 & df2$Hour2<=19) ~ 'Evening',
                         (df2$Hour2>=12 & df2$Hour2<=16) ~ 'Mid')

#exclusion of vaccines given between 10pm to 8am###
df2$nightVac <- case_when(df2$Hour2 <8 | df2$Hour2 > 22 ~ 'nightVacc')

dfnight <- df2 %>% drop_na(nightVac)
## censoring dose 4
# first set a variable 0 if date 4 is before the COVID test and 1×‟ if
its after
df2$isDat4 <- case_when(as.Date(df2$Date4) - as.Date(df2$Sample_date) < 0
~ 1,
                        TRUE ~ 0
                        #as.Date(df2$Date4) - as.Date(df2$Sample_date) <0
~ 0
)

#df2 %>% select(Date4, isDat4, Sample_date)

df2$pos_covid <- case_when((df2$IS_POSITIVE_CD==2 |
df2$IS_POSITIVE_CD==21 & df2$isDat4==0) ~ 1,

                           TRUE ~ 0
)


df2$endDate <- "2022-04-26"    ###Date of data extraction####
df2$follow_up <- as.Date(df2$endDate) - as.Date(df2$Date2)

df2$pos_covid <- as.factor(df2$pos_covid)
summary(df2$pos_covid) # only positive

df2_clean <- df2 %>% drop_na(hourBin)
```

```
hist(df2_clean$Age1)

df2_clean$diff2 <- as.Date(df2_clean$Date2) - as.Date(df2_clean$Date1)
df2_clean$diff3 <- as.Date(df2_clean$Date3) - as.Date(df2_clean$Date2)
hist(as.numeric(df2_clean$follow_up))

## organize comorbidity and labels

df2_clean$diabetes_bin <- case_when(df2_clean$STATUS_IN_DIABETIC == 1 ~
1,
                                    df2_clean$STATUS_IN_DIABETIC == 0 ~
0)


df2_clean$HTN_bin <- case_when(df2_clean$STATUS_IN_BLOODPRESURE == 1 ~ 1,
                               df2_clean$STATUS_IN_BLOODPRESURE == 0 ~ 0)

df2_clean$CKD_bin <- case_when(df2_clean$STATUS_IN_CKD == 1 ~ 1,

                               df2_clean$STATUS_IN_CKD == 0 ~ 0)


df2_clean$immunosup_bin <- case_when(df2_clean$IMMUNOSUPPRESSION_Status
== 1 ~ 1,

                                     df2_clean$IMMUNOSUPPRESSION_Status
== 0 ~ 0)


df2_clean$dialisis_bin <- case_when(df2_clean$STATUS_IN_DIALIZA==1 ~ 1,
                                    df2_clean$STATUS_IN_DIALIZA==0 ~ 0)

df2_clean$astma <- case_when(df2_clean$Asthma_ever == 1 ~ 1,
                             df2_clean$Asthma_ever == 0 ~ 0)

df2_clean$nerologic <- case_when(df2_clean$Neurologic_disease_ever == 1 ~
1,
                                 df2_clean$Neurologic_disease_ever == 0 ~
0)

df2_clean$obesity <- case_when (df2_clean$BMI <= 30 ~ 0,
                                df2_clean$BMI > 30 ~ 1)



# cancer IND
# summarizing comorbidities
df2_clean <- df2_clean %>% mutate(sumCom =
diabetes_bin+HTN_bin+CKD_bin+immunosup_bin+dialisis_bin+astma
                                  +nerologic+Cancer_Diag_Last_5_Yrs_Ind +
obesity + Heart_Disease_ever)

summary(df2_clean$sumCom)
```

```
# group comorbidities and labeling
df2_clean <- df2_clean %>% mutate(
  comBins = case_when(sumCom > 4 ~ '4+',
                      sumCom <=4 & sumCom>=1 ~ '1-4',
                      TRUE ~ '0'),
  diabetes = as.factor(case_when(diabetes_bin==1 ~ 'Yes',
                                 TRUE ~ 'No')),
  HTN = case_when(HTN_bin==1 ~ 'Yes',TRUE ~ 'No'),
  CKD = case_when(CKD_bin==1 ~ 'Yes', TRUE ~ 'No'),

  immunosup = case_when(immunosup_bin == 1 ~ 'Yes', TRUE ~ 'No'),

  dialisis = case_when(dialisis_bin== 1 ~ 'Yes', TRUE ~ 'No'),
  astamaL = case_when(astma == 1 ~ 'Yes', TRUE ~ 'No'),
  cancer = case_when(Cancer_Diag_Last_5_Yrs_Ind == 1 ~ 'Yes', TRUE ~
'No'),
  neuro = case_when(nerologic == 1 ~'Yes', TRUE ~ 'No'),
  obese = case_when(obesity == 1 ~'Yes', TRUE ~ 'No'),
  heart = case_when(Heart_Disease_ever == 1 ~ 'Yes', TRUE ~ 'No')
)

# choose relevant variable


dfSort <- dplyr::select(df2_clean, Age1, CUSTOMER_SEX_CODE, comBins,
diabetes, HTN,neuro,
                        CKD, immunosup, dialisis, astamaL, cancer,
hourBin,obese, heart,
                        diff3)

# build summary table
theme_gtsummary_journal(journal="jama")
theme_gtsummary_compact()
t2 <- tbl_summary(
  dfSort,
  by = hourBin,
  label = list(Age1 ~ "Age",
               CUSTOMER_SEX_CODE ~ "Sex",
               comBins ~ "Comorbidities",
               diabetes ~ "Diabetic",
               HTN ~ "Blood Pressure",
               neuro ~ "Neurological Disease",
               CKD ~ "CKD",
               immunosup ~ "Immuno Suppress",
               dialisis ~"Dialysis",
               astamaL ~ "Asthma",
               cancer ~ "Cancer last 5yrs",
               obese ~ "Obesity",
               heart ~ "Heart Disease",
               diff3 ~ "Time between vaccinations 2-3"
               #follow_up ~ "Follow-Up"

  ),
```

```
  statistic = list(all_continuous() ~ "{median} ({p25}, {p75})"),
missing="no",

) %>%  modify_header(label="**Variable**") %>% bold_labels()  %>% add_p()

t2

# turn to word
as_gt(t2) %>%  gt::gtsave('Table1R.rtf')

# descriptive analysis for dose 3#
df3 <- read.csv('general3.csv')

df3$hourBin <- case_when((df3$Hour3>=8 & df3$Hour3<=11) ~ 'Morning',
                         (df3$Hour3>=16 & df3$Hour3<=19) ~ 'Evening',
                         (df3$Hour3>=12 & df3$Hour3<=16) ~ 'Mid'
)

df3$hourBin <- factor(df3$hourBin)
df3_clean <- df3 %>% drop_na(hourBin)# %>% filter(Age1>=16) # total data
within the morning/evening definition is 550163

df3_clean$pos_covid <- case_when((df3_clean$IS_POSITIVE_CD==2 |
df3_clean$IS_POSITIVE_CD==21) ~ 1,
                                 TRUE ~ 0
)
summary(as.factor(df3_clean$pos_covid))

# organize data (using a function)
dfSort3 <- organizeD(df3_clean)


# table dose 3
theme_gtsummary_journal(journal="jama")
theme_gtsummary_compact()
t3 <- tbl_summary(
  dfSort3,
  by = hourBin,
  label = list(Age1 ~ "Age",
               CUSTOMER_SEX_CODE ~ "Sex",
               comBins ~ "Comorbidities",
               diabetes ~ "Diabetic",
               HTN ~ "Blood Pressure",
               neuro ~ "Neurological Disease",
               CKD ~ "CKD",
               immunosup ~ "Immuno Suppress",
               dialisis ~"Dialysis",
               astamaL ~ "Asthma",
               cancer ~ "Cancer last 5yrs",
               obese ~ "Obesity",
               heart ~ "Heart Disease"


  ),
```

```
  statistic = list(all_continuous() ~ "{median} ({p25}, {p75})"),
missing="no",

) %>%   modify_header(label="**Variable**") %>% bold_labels() %>% add_p()

t3

tbl_merge(list(t2,t3), tab_spanner = c('Dose2', 'Dose3'))


# descriptive analysis for dose 4#
df4 <- read.csv('general4.csv')

df4$hourBin <- case_when((df4$Hour4>=8 & df4$Hour4<=11) ~ 'Morning',
                         (df4$Hour4>=16 & df4$Hour4<=19) ~ 'Evening',
                         (df4$Hour4>=12 & df4$Hour4<=16) ~ 'Mid'
)

df4$hourBin <- factor(df4$hourBin)
df4_clean <- df4 %>% drop_na(hourBin)# %>% filter(Age1 >=16) # total data
within the morning/evening definition is 550163

df4_clean$pos_covid <- case_when((df4_clean$IS_POSITIVE_CD==2 |
df4_clean$IS_POSITIVE_CD==21) ~ 1,
                                 TRUE ~ 0
)
summary(as.factor(df4_clean$pos_covid))

dfSort4 <- organizeD(df4_clean)

# table dose 4
# table dose 3
theme_gtsummary_journal(journal="jama")
theme_gtsummary_compact()
t4 <- tbl_summary(
  dfSort4,
  by = hourBin,
  label = list(Age1 ~ "Age",
               CUSTOMER_SEX_CODE ~ "Sex",
               comBins ~ "Comorbidities",
               diabetes ~ "Diabetic",
               HTN ~ "Blood Pressure",
               neuro ~ "Neurological Disease",
               CKD ~ "CKD",
               immunosup ~ "Immuno Suppress",
               dialisis ~"Dialysis",
               astamaL ~ "Asthma",
               cancer ~ "Cancer last 5yrs",
               obese ~ "Obesity",
               heart ~ "Heart Disease"


  ),
```

```
  statistic = list(all_continuous() ~ "{median} ({p25}, {p75})"),
missing="no",

) %>%   modify_header(label="**Variable**") %>% bold_labels()  %>%
add_p()

# merge all three tables
library(flextable)
tbl_merge(list(t2,t3, t4), tab_spanner = c('Dose2', 'Dose3', 'Dose4'))
%>%
  as_flex_table() %>%
flextable::save_as_docx(path="Tabl1CombinedR_Feb2023.docx")


### Univariate Survival analysis
library(survival)
library(survminer)
library(ggfortify)

df2_clean$endDate = "2022-04-26"    ####Date of data extraction#####
df2_clean$Sample_date_censor <- if_else(is.na(df2_clean$Sample_date),
"2022-04-26",df2_clean$Sample_date)

baseDate <- as.Date(df2_clean$Date2) + 6 # basedate is set for 6 days
after second vaccine
dateDiff <- as.Date(df2_clean$Sample_date_censor) - baseDate

hist(as.numeric(dateDiff))

df2_clean$dateDiff <- as.numeric(dateDiff)
df2_clean <- subset(df2_clean, df2_clean$dateDiff > 0 ) # remove
infections before basedate


# by time of vaccination
model1 <- Surv(time = df2_clean$dateDiff, event =
as.numeric(df2_clean$pos_covid))
fit1 <- survfit(model1 ~ hourBin , data=df2_clean)
#setEPS()
cairo_ps('FigureSHourBinR2.eps')
autoplot(fit1, censor = F) + theme_bw() + theme(panel.border =
element_blank(),
                                                panel.grid.major =
element_blank(),
                                                panel.grid.minor =
element_blank()) +  ggtitle("Am/PM Survival")# +
#ggsave('FigureSHourBin.eps', device = cairo_pdf)
dev.off()

# grab group and survival percentage
# first 466 is evening, other is morning
fit1$strata
surv_fit1 <- fit1$surv
```

```
standard_error_fit1 <- fit1$std.err
time_fit1 <- fit1$time
group = c(rep('evening', 467), rep('Mid', 466), rep('Morning', 465))
fit1_table <- data.frame(time_fit1, surv_fit1, standard_error_fit1,
group)
# save
write.csv(fit1_table, "am_pm_survival_midR.csv")


# remove the midday for the next univariate
dfNoMid <- df2_clean %>% filter(hourBin!='Mid')

model2 <- Surv(time = df2_clean$dateDiff, event =
as.numeric(df2_clean$pos_covid))
#by gender
fit2 <- survfit(model2 ~ CUSTOMER_SEX_CODE + hourBin , data=df2_clean)
cairo_ps('FigureGenderR2_dose4.eps')
autoplot(fit2, censor = F) +theme_bw() + theme(panel.border =
element_blank(),
                                               panel.grid.major =
element_blank(),
                                               panel.grid.minor =
element_blank())+ ggtitle("Gender Survival")
dev.off()

fit2$strata
surv_fit2 <- fit2$surv
standard_error_fit2 <- fit2$std.err
time_fit2 <- fit2$time
group = c(rep('evening_female', 465), rep('Mid_female', 465),
rep('morning_female', 465),
          rep('evening_male', 467),rep('Mid_male', 466),
rep('morning_male', 465))
fit2_table <- data.frame(time_fit2, surv_fit2, standard_error_fit2,
group)

write.csv(fit2_table, "am_pm_m_f_midR.csv")


# by comorbidity

fit3 <- survfit(model2 ~ comBins + hourBin , data=df2_clean)
#postscript('FigureComorbidities.eps')
cairo_ps('FigureComorbiditiesR2_dose4.eps')
autoplot(fit3, censor = F) +theme_bw() + theme(panel.border =
element_blank(),
                                               panel.grid.major =
element_blank(),
                                               panel.grid.minor =
element_blank()) + ggtitle("Comorbidity Survival")# +
ggsave("figureComb.eps")
dev.off()

# output
```

```
fit3$strata
surv_fit3 <- fit3$surv
standard_error_fit3 <- fit3$std.err
time_fit3 <- fit3$time
group = c(rep('evening_0com', 467),rep('mid_0cm', 466),
rep('morning_0comb', 465),
          rep('evening_1_4comb', 465), rep('mid_1_4comb', 465),
rep('morning_1_4comb', 465),
          rep('evening_4pluscomb', 290),rep('mid_4pluscomb', 318),
rep('morning_4pluscomb', 348))
fit3_table <- data.frame(time_fit3, surv_fit3, standard_error_fit3,
group)

write.csv(fit3_table, "am_pm_comorbidities_midR.csv")



# by age (young/old)
df2_clean$Age2_old <- case_when(df2_clean$Age2<30 ~ '12-30',
                                (df2_clean$Age2 >=30 & df2_clean$Age2<60)
~ '30-60',
                                df2_clean$Age2 > 60 ~ '60+')

fit4 <- survfit(model2 ~ Age2_old + hourBin , data=df2_clean)
cairo_ps('FigureAgeR2_dose4.eps')
autoplot(fit4, censor = F) +theme_bw() + theme(panel.border =
element_blank(),
                                               panel.grid.major =
element_blank(),
                                               panel.grid.minor =
element_blank()) + ggtitle("Age Survival")# + ggsave("figureComb.eps")
dev.off()

# output
fit4$strata
surv_fit4 <- fit4$surv
standard_error_fit4 <- fit4$std.err
time_fit4 <- fit4$time
group = c(rep('evening_12_30', 466), rep('mid_12_30', 465),
rep('morning_12_30', 465),
          rep('evening_30_60', 462),rep('mid_30_60', 461),
rep('morning_30_60', 465),
          rep('evening_60plus', 438),rep('mid_60plus', 453),
rep('morning_60plus', 456))
fit4_table <- data.frame(time_fit4, surv_fit4, standard_error_fit4,
group)

write.csv(fit4_table, "am_pm_age_midR.csv")


df1 <- read.csv('general.csv')


df1 <- df1 %>% filter(Age1 >=12)
```

```
# total people enrolled before study period
df1Nd <- df1 %>% filter(SECOND_VACCINE_IND==1) # 1,515,574

nrow(Population) - nrow(df1Nd)


# no documented covid prior dose 2
df1Nd$baseDate <- as.Date(df1Nd$Date2)
df1Nd$Sample_date_censor <- if_else (is.na(df1Nd$Sample_date), "2022-04-
26",df1Nd$Sample_date)
df1Nd$dateDiff <- as.Date(df1Nd$Sample_date_censor) -
as.Date(df1Nd$baseDate)
df1Nd$dateDiff <- as.numeric(df1Nd$dateDiff)
hist(df1Nd$dateDiff)
# no COVID prior dose 2
df1NC <- df1Nd %>% filter(dateDiff >= 0) # 1,515,910

diagnostic_test <- read.table ("Circadian_Clock_All_PCR.csv",sep = "\t",
header = TRUE)

diagnostic_test$timeSample <-
as.numeric(substr(diagnostic_test$SAMPLE_EXECUTION_DATE,11, 13))

# merge together
dfSample <- left_join(df1NC, diagnostic_test, "InternalPatID" )
dfSample_c <- dfSample %>% filter(!is.na(timeSample)) # 3,930,309

# difference
nrow(dfSample) - nrow(dfSample_c) # 810760

# dose 3
dfSample_c3 <- dfSample_c %>% filter(THIRD_VACCINE_IND==1) # 3,259,718
# difference
nrow(dfSample_c) - nrow(dfSample_c3) # 670,591

# dose 4

dfSample_c4 <- dfSample_c3 %>% filter(FOURTH_VACCINE_IND==1) # 643,289
# difference
nrow(dfSample_c3) - nrow(dfSample_c4) # 2,616,429


# df4

## number of participants in the model by age grou
# take the df2_clean from dose 2 landmark script
df2_clean$ageCat <- case_when(df2_clean$Age2 <30 ~ "12-30",
                              df2_clean$Age2 >=30 & df2_clean$Age2 <60 ~
'30-60',
                              df2_clean$Age2 >=60 ~ '60+')

df2_clean$ageCat <- as.factor(df2_clean$ageCat)
summary(as.factor(df2_clean$ageCat))
```

```
df2_clean %>% filter(hourBin=='Morning') %>% group_by(ageCat)
%>%summarise(count = n())

# Dose 3 model

summary(as.factor(df3_clean$FOURTH_VACCINE_IND))

df3_clean$ageCat <- case_when(df3_clean$Age2 <30 ~ "12-30",
                              df3_clean$Age2 >=30 & df3_clean$Age2 <60 ~
'30-60',
                              df3_clean$Age2 >=60 ~ '60+')

df3_clean$ageCat <- as.factor(df3_clean$ageCat)
summary(as.factor(df3_clean$ageCat))
df3_clean %>% filter(hourBin=='Morning') %>% group_by(ageCat)
%>%summarise(count = n())

df2 <- read.csv('general2.csv')

df2$hourBin <- case_when((df2$Hour1>=8 & df2$Hour1<=11 & df2$Hour2>=8 &
df2$Hour2<=11) ~ 'Morning',
                         (df2$Hour1>=16 & df2$Hour1<=19 & df2$Hour2>=16 &
df2$Hour2<=19) ~ 'Evening',
)

df2$pos_covid <- case_when((df2$IS_POSITIVE_CD==2 |
df2$IS_POSITIVE_CD==21) ~ 1,
                           TRUE ~ 0
)

df2$endDate <- "2022-04-26"
df2$follow_up <- as.Date(df2$endDate) - as.Date(df2$Date2)

df2$pos_covid <- as.factor(df2$pos_covid)
summary(df2$pos_covid) # only positive

df2_clean <- df2 %>% drop_na(hourBin) #%>% filter(Age1>=16) # total data
within the morning/evening definition is 158019
hist(df2_clean$Age1)

df2_clean$diff2 <- as.Date(df2_clean$Date2) - as.Date(df2_clean$Date1)
df2_clean$diff3 <- as.Date(df2_clean$Date3) - as.Date(df2_clean$Date2)
hist(as.numeric(df2_clean$follow_up))

## organize comorbidity and labels

df2_clean$diabetes_bin <- case_when(df2_clean$STATUS_IN_DIABETIC == 1 ~
1,
                                    df2_clean$STATUS_IN_DIABETIC == 0 ~
0)


df2_clean$HTN_bin <- case_when(df2_clean$STATUS_IN_BLOODPRESURE == 1 ~ 1,
                               df2_clean$STATUS_IN_BLOODPRESURE == 0 ~ 0)
```

```
df2_clean$CKD_bin <- case_when(df2_clean$STATUS_IN_CKD == 1 ~ 1,

                               df2_clean$STATUS_IN_CKD == 0 ~ 0)


df2_clean$immunosup_bin <- case_when(df2_clean$IMMUNOSUPPRESSION_Status
== 1 ~ 1,

                                     df2_clean$IMMUNOSUPPRESSION_Status
== 0 ~ 0)


df2_clean$dialisis_bin <- case_when(df2_clean$STATUS_IN_DIALIZA==1 ~ 1,
                                    df2_clean$STATUS_IN_DIALIZA==0 ~ 0)

df2_clean$astma <- case_when(df2_clean$Asthma_ever == 1 ~ 1,
                             df2_clean$Asthma_ever == 0 ~ 0)

df2_clean$nerologic <- case_when(df2_clean$Neurologic_disease_ever == 1 ~
1,
                                 df2_clean$Neurologic_disease_ever == 0 ~
0)

df2_clean$obesity <- case_when (df2_clean$BMI <= 30 ~ 0,
                                df2_clean$BMI > 30 ~ 1)



# cancer IND
# summarizing comorbidities
df2_clean <- df2_clean %>% mutate(sumCom =
diabetes_bin+HTN_bin+CKD_bin+immunosup_bin+dialisis_bin+astma
                                  +nerologic+Cancer_Diag_Last_5_Yrs_Ind +
obesity + Heart_Disease_ever)

summary(df2_clean$sumCom)

# group comorbidities and labeling
df2_clean <- df2_clean %>% mutate(
  comBins = case_when(sumCom > 4 ~ '4+',
                      sumCom <=4 & sumCom>=1 ~ '1-4',
                      TRUE ~ '0'),
  diabetes = as.factor(case_when(diabetes_bin==1 ~ 'Yes',
                                 TRUE ~ 'No')),
  HTN = case_when(HTN_bin==1 ~ 'Yes',TRUE ~ 'No'),
  CKD = case_when(CKD_bin==1 ~ 'Yes', TRUE ~ 'No'),

  immunosup = case_when(immunosup_bin == 1 ~ 'Yes', TRUE ~ 'No'),

  dialisis = case_when(dialisis_bin== 1 ~ 'Yes', TRUE ~ 'No'),
  astamaL = case_when(astma == 1 ~ 'Yes', TRUE ~ 'No'),
  cancer = case_when(Cancer_Diag_Last_5_Yrs_Ind == 1 ~ 'Yes', TRUE ~
'No'),
```

```
  neuro = case_when(nerologic == 1 ~'Yes', TRUE ~ 'No'),
  obese = case_when(obesity == 1 ~'Yes', TRUE ~ 'No'),
  heart = case_when(Heart_Disease_ever == 1 ~ 'Yes', TRUE ~ 'No')
)

# choose relevant variable


dfSort <- dplyr::select(df2_clean, Age1, CUSTOMER_SEX_CODE, comBins,
diabetes, HTN,neuro,
                        CKD, immunosup, dialisis, astamaL, cancer,
hourBin,obese, heart,
                        diff3)

# build summary table
theme_gtsummary_journal(journal="jama")
theme_gtsummary_compact()
t2 <- tbl_summary(
  dfSort,
  by = hourBin,
  label = list(Age1 ~ "Age",
               CUSTOMER_SEX_CODE ~ "Sex",
               comBins ~ "Comorbidities",
               diabetes ~ "Diabetic",
               HTN ~ "Blood Pressure",
               neuro ~ "Neurological Disease",
               CKD ~ "CKD",
               immunosup ~ "Immuno Suppress",
               dialisis ~"Dialysis",
               astamaL ~ "Asthma",
               cancer ~ "Cancer last 5yrs",
               obese ~ "Obesity",
               heart ~ "Heart Disease",
               diff3 ~ "Time between vaccinations 2-3"
               #follow_up ~ "Follow-Up"

  ),

  statistic = list(all_continuous() ~ "{median} ({sd})"), missing="no",

) %>%  modify_header(label="**Variable**") %>% bold_labels() %>% add_p()

t2

# turn to word
as_gt(t2) %>%  gt::gtsave('Table1.rtf')

# dose 3
df3 <- read.csv('general3.csv')

df3$hourBin <- case_when((df3$Hour3>=8 & df3$Hour3<=11) ~ 'Morning',
                         (df3$Hour3>=16 & df3$Hour3<=19) ~ 'Evening',
)
```

```
df3$hourBin <- factor(df3$hourBin)
df3_clean <- df3 %>% drop_na(hourBin)# %>% filter(Age1>=16) # total data
within the morning/evening definition is 550163

df3_clean$pos_covid <- case_when((df3_clean$IS_POSITIVE_CD==2 |
df3_clean$IS_POSITIVE_CD==21) ~ 1,
                                  TRUE ~ 0
)
summary(as.factor(df3_clean$pos_covid))

# organize data (using a function)
dfSort3 <- organizeD(df3_clean)


# table dose 3
theme_gtsummary_journal(journal="jama")
theme_gtsummary_compact()
t3 <- tbl_summary(
  dfSort3,
  by = hourBin,
  label = list(Age1 ~ "Age",
               CUSTOMER_SEX_CODE ~ "Sex",
               comBins ~ "Comorbidities",
               diabetes ~ "Diabetic",
               HTN ~ "Blood Pressure",
               neuro ~ "Neurological Disease",
               CKD ~ "CKD",
               immunosup ~ "Immuno Suppress",
               dialisis ~"Dialysis",
               astamaL ~ "Asthma",
               cancer ~ "Cancer last 5yrs",
               obese ~ "Obesity",
               heart ~ "Heart Disease"


  ),

  statistic = list(all_continuous() ~ "{median} ({sd})"), missing="no",

) %>%   modify_header(label="**Variable**") %>% bold_labels()

t3

tbl_merge(list(t2,t3), tab_spanner = c('Dose2', 'Dose3'))


# dose 4
df4 <- read.csv('general4.csv')

df4$hourBin <- case_when((df4$Hour4>=8 & df4$Hour4<=11) ~ 'Morning',
                         (df4$Hour4>=16 & df4$Hour4<=19) ~ 'Evening',
)

df4$hourBin <- factor(df4$hourBin)
```

```
df4_clean <- df4 %>% drop_na(hourBin)# %>% filter(Age1 >=16) # total data
within the morning/evening definition is 550163

df4_clean$pos_covid <- case_when((df4_clean$IS_POSITIVE_CD==2 |
df4_clean$IS_POSITIVE_CD==21) ~ 1,
                                  TRUE ~ 0
)
summary(as.factor(df4_clean$pos_covid))

dfSort4 <- organizeD(df4_clean)

# table dose 4
# table dose 3
theme_gtsummary_journal(journal="jama")
theme_gtsummary_compact()
t4 <- tbl_summary(
  dfSort4,
  by = hourBin,
  label = list(Age1 ~ "Age",
               CUSTOMER_SEX_CODE ~ "Sex",
               comBins ~ "Comorbidities",
               diabetes ~ "Diabetic",
               HTN ~ "Blood Pressure",
               neuro ~ "Neurological Disease",
               CKD ~ "CKD",
               immunosup ~ "Immuno Suppress",
               dialisis ~"Dialysis",
               astamaL ~ "Asthma",
               cancer ~ "Cancer last 5yrs",
               obese ~ "Obesity",
               heart ~ "Heart Disease"


  ),

  statistic = list(all_continuous() ~ "{median} ({sd})"), missing="no",

) %>%   modify_header(label="**Variable**") %>% bold_labels()

# merge all three tables
library(flextable)
tbl_merge(list(t2,t3, t4), tab_spanner = c('Dose2', 'Dose3', 'Dose4'))
%>%
  as_flex_table() %>% flextable::save_as_docx(path="Tabl1CombinedR.docx")


#######multivariate analysis#####

library(tidyverse)
library(dplyr)
library(tidyr)

# analytic approach number 1: by casual inference#
```

```
df <- read.csv('general2.csv')


## mutate to add time to dose (3 and 4)
df <- df[,3:97]

df2 <- df %>% mutate(
  t0 = Date2,
  diff2_3 = as.Date(Date3) - as.Date(t0),
  diff2_4 =as.Date(Date4) - as.Date(t0)
)


df2$hourBin <- case_when((df2$Hour2>=8 & df2$Hour2<=11) ~ 'Morning',
                         (df2$Hour2>=16 & df2$Hour2<=19) ~ 'Evening',
                         (df2$Hour2>=12 & df2$Hour2<=16) ~ 'Mid'
)

df2$pos_covid <- case_when((df2$IS_POSITIVE_CD==2 |
df2$IS_POSITIVE_CD==21) ~ 1,
                           TRUE ~ 0
)

# censoring people who received dose 4
# first set a variable 0 if date 4 is before the COVID test and 1 if its
after
df2$isDat4 <- case_when(as.Date(df2$Date4) - as.Date(df2$Sample_date) < 0
~ 1,
                        TRUE ~ 0
                        #as.Date(df2$Date4) - as.Date(df2$Sample_date) <0
~ 0
)

#df2 %>% select(Date4, isDat4, Sample_date)

summary(as.factor(df2$isDat4))
hist(df2$isDat4)
df2$pos_covid <- case_when((df2$IS_POSITIVE_CD==2 |
df2$IS_POSITIVE_CD==21 & df2$isDat4==0) ~ 1,

                           TRUE ~ 0
)

df2$endDate <- "2022-04-26"
df2$follow_up <- as.Date(df2$endDate) - as.Date(df2$Date2)

df2$pos_covid <- as.factor(df2$pos_covid)



cleanDat <- function(df2) {
  df2_clean <- df2 %>% drop_na(hourBin)

  df2_clean$Age2_old <- case_when(df2_clean$Age2 <= 60 ~ 0,
```

```
                                df2_clean$Age2 > 60 ~ 1)

  df2_clean$diabetes_bin <- case_when(df2_clean$STATUS_IN_DIABETIC == 1 ~
1,
                                      df2_clean$STATUS_IN_DIABETIC == 0 ~
0)


  df2_clean$HTN_bin <- case_when(df2_clean$STATUS_IN_BLOODPRESURE == 1 ~
1,
                                 df2_clean$STATUS_IN_BLOODPRESURE == 0 ~
0)

  df2_clean$CKD_bin <- case_when(df2_clean$STATUS_IN_CKD == 1 ~ 1,

                                 df2_clean$STATUS_IN_CKD == 0 ~ 0)


  df2_clean$immunosup_bin <- case_when(df2_clean$IMMUNOSUPPRESSION_Status
== 1 ~ 1,

                                       df2_clean$IMMUNOSUPPRESSION_Status
== 0 ~ 0)


  df2_clean$dialisis_bin <- case_when(df2_clean$STATUS_IN_DIALIZA==1 ~ 1,
                                      df2_clean$STATUS_IN_DIALIZA==0 ~ 0)

  df2_clean$astma <- case_when(df2_clean$Asthma_ever == 1 ~ 1,
                               df2_clean$Asthma_ever == 0 ~ 0)

  df2_clean$nerologic <- case_when(df2_clean$Neurologic_disease_ever == 1
~ 1,
                                   df2_clean$Neurologic_disease_ever == 0
~ 0)

  df2_clean$obesity <- case_when (df2_clean$BMI <= 30 ~ 0,
                                  df2_clean$BMI > 30 ~ 1)


  # set as factors

  df2_clean$Age2_old <- as.factor(df2_clean$Age2_old)
  df2_clean$STATUS_IN_DIABETIC <- as.factor(df2_clean$STATUS_IN_DIABETIC)
  df2_clean$diabetes_bin <- as.factor(df2_clean$diabetes_bin)
  df2_clean$obesity <- as.factor(df2_clean$obesity)
  df2_clean$HTN_bin <- as.factor(df2_clean$HTN_bin)
  df2_clean$CKD_bin <- as.factor(df2_clean$CKD_bin)
  df2_clean$immunosup_bin <- as.factor(df2_clean$immunosup_bin)
  df2_clean$gender <- as.factor(df2_clean$CUSTOMER_SEX_CODE)

  return(df2_clean)
}
```

```
# change to long format (each subject has three lines (dose 2,3,4))
#df_long <- df2_clean %>% gather(cond_diff, diff, diff2_3:diff2_4)



### Univariate Survival analysis
library(survival)
library(survminer)
library(ggfortify)


model1 <- coxph (Surv(follow_up, pos_covid) ~  gender + hourBin +
                   diabetes_bin + Age2_old + obesity + CKD_bin +HTN_bin
+diff2_3,
                 cluster = InternalPatID, id = InternalPatID,
                 data=df2_clean)

summary(model1)

model1 %>% gtsummary::tbl_regression(exp=T)


# test permutation of all options of hourbin (i.e., Am:PM, AM:AM etc/)

# generate specific hourBin
df2$hourBinP <- case_when((df2$Hour1>=8 & df2$Hour1<=11 & df2$Hour2>=8 &
df2$Hour2<=11) ~ 'AMAM',
                         (df2$Hour1>=8 & df2$Hour1<=11 & df2$Hour2>=16 &
df2$Hour2<=19) ~ 'AMPM',
                         (df2$Hour1>=16 & df2$Hour1<=19 & df2$Hour2>=8 &
df2$Hour2<=11) ~ 'PMAM',
                         (df2$Hour1>=16 & df2$Hour1<=19 & df2$Hour2>=16
& df2$Hour2<=19) ~ 'PMPM'
)


model2 <- coxph (Surv(follow_up, pos_covid) ~  gender + hourBinP +
                   diabetes_bin + Age2_old + obesity + CKD_bin +HTN_bin
+diff2_3,
                 cluster = InternalPatID, id = InternalPatID,
                 data=df2_clean)

summary(model2)
model2 %>% gtsummary::tbl_regression(exp=T)


# compare morning to evening only
df2$hourBin <- case_when((df2$Hour2>=8 & df2$Hour2<=11) ~ 'AM',
                         (df2$Hour2>=16 & df2$Hour2<=19) ~ 'PM',

)

# go back to run all data cleaning and factorization
df2_cleanAMPM <- cleanDat(df2) # clean data
```

```
model3 <- coxph (Surv(follow_up, pos_covid) ~  gender + hourBinP +
                   diabetes_bin + Age2_old + obesity + CKD_bin +HTN_bin
+diff2_3,
                 cluster = InternalPatID, id = InternalPatID,
                 data=df2_cleanAMPM)

summary(model3)
model3 %>% gtsummary::tbl_regression(exp=T)


###Analytic approach number 2: Cox regression with time-dependent
variables by landmark model

df2 <- read.csv('general2.csv')

df2$hourBin <- case_when((df2$Hour1>=8 & df2$Hour1<=11 & df2$Hour2>=8 &
df2$Hour2<=11) ~ 'Morning',
                         (df2$Hour1>=16 & df2$Hour1<=19 & df2$Hour2>=16 &
df2$Hour2<=19) ~ 'Evening',
)
df2$hourBin

df2_clean <- df2 %>% drop_na(hourBin) # total data within the
morning/evening definition is 550163
df2_clean$FOURTH_VACCINE_IND
df2_clean <- df2_clean %>% filter(FOURTH_VACCINE_IND == "0")  ##with this
approach we excluded the second booster dose (see methods section)###
df2_clean$FOURTH_VACCINE_IND
summary (as.factor(df2_clean$hourBin))
df2_clean$pos_covid <- case_when((df2_clean$IS_POSITIVE_CD==2 |
df2_clean$IS_POSITIVE_CD==21) ~ 1,
                                 TRUE ~ 0)

summary(as.factor(df2_clean$pos_covid))



baseDate <- as.Date(df2_clean$Date2) + 6
df2_clean$Sample_date_censor <- if_else (is.na(df2_clean$Sample_date),
"2022-04-26",df2_clean$Sample_date)
dateDiff <- as.Date(df2_clean$Sample_date_censor) - baseDate
hist(as.numeric(dateDiff))


sort (df2_clean$Date3)
df2_clean$landmark3 <- as.Date("2021-07-04") #Date for the first dose 3
that was given in this cohort#
df2_clean$endDate <- as.Date("2022-04-26")
df2_clean$diff3 <- as.Date(df2_clean$Sample_date) -
as.Date(df2_clean$landmark3)
hist (as.numeric(df2_clean$diff3))
df2_clean$THIRD_VACCINE_IND <- as.factor(df2_clean$THIRD_VACCINE_IND)
```

```
df2_clean$Age2_old <- case_when(df2_clean$Age2 <= 60 ~ 0,
                                df2_clean$Age2 > 60 ~ 1)

df2_clean$Age2_old <- as.factor(df2_clean$Age2_old)


df2_clean$obesity <- case_when (df2_clean$BMI <= 30 ~ 0,
                                df2_clean$BMI > 30 ~ 1)
df2_clean$obesity <- if_else(is.na((df2_clean$obesity)), 0,
df2_clean$obesity)
df2_clean$obesity <- as.factor(df2_clean$obesity)

df2_clean$diabetes_bin <- case_when(df2_clean$STATUS_IN_DIABETIC == 1 ~
1,
                                    df2_clean$STATUS_IN_DIABETIC == 0 ~
0)
df2_clean$diabetes_bin
df2_clean$diabetes_bin <- if_else(is.na((df2_clean$diabetes_bin)), 0,
df2_clean$diabetes_bin)
df2_clean$diabetes_bin
df2_clean$HTN_bin <- case_when(df2_clean$STATUS_IN_BLOODPRESURE == 1 ~ 1,
                               df2_clean$STATUS_IN_BLOODPRESURE == 0 ~ 0)
df2_clean$HTN_bin
df2_clean$HTN_bin <- if_else(is.na((df2_clean$HTN_bin)), 0,
df2_clean$HTN_bin)
df2_clean$CKD_bin <- case_when(df2_clean$STATUS_IN_CKD == 1 ~ 1,

                               df2_clean$STATUS_IN_CKD == 0 ~ 0)
df2_clean$CKD_bin <- if_else(is.na((df2_clean$CKD_bin)), 0,
df2_clean$CKD_bin)

df2_clean$immunosup_bin <- case_when(df2_clean$IMMUNOSUPPRESSION_Status
== 1 ~ 1,

                                     df2_clean$IMMUNOSUPPRESSION_Status
== 0 ~ 0)
df2_clean$immunosup_bin <- if_else(is.na((df2_clean$immunosup_bin)), 0,
df2_clean$immunosup_bin)

df2_clean$Age2_old <- as.factor(df2_clean$Age2_old)
df2_clean$STATUS_IN_DIABETIC <- as.factor(df2_clean$STATUS_IN_DIABETIC)
df2_clean$diabetes_bin <- as.factor(df2_clean$diabetes_bin)
df2_clean$obesity <- as.factor(df2_clean$obesity)
df2_clean$HTN_bin <- as.factor(df2_clean$HTN_bin)
df2_clean$CKD_bin <- as.factor(df2_clean$CKD_bin)
df2_clean$immunosup_bin <- as.factor(df2_clean$immunosup_bin)



###bootstrap###
library(boot)
boot.cox <- function(df, indices) {
  samples <- df[indices, ]
```

```
  fit <-  coxph (Surv(dateDiff, pos_covid) ~ THIRD_VACCINE_IND + hourBin
+ Age2_old + obesity + HTN_bin + SES_bin + diabetes_bin + CKD_bin
+immunosup_bin,
                 subset = diff3 > 0 | is.na(diff3),
                 data=samples)
  coef(fit)
}

model.boot <- boot(df2_clean, boot.cox, 2000, parallel = 'snow')
#boot.ci(model.boot, index=2)
model.boot$t0
# grab hourBIN (t0=2)
quantile(model.boot$t[,2],probs = c(0.025, 0.975))

###check the model###
results <- coxph (Surv(dateDiff, pos_covid) ~ THIRD_VACCINE_IND + hourBin
+ Age2 + HTN_bin + obesity + gender + diabetes_bin + CKD_bin,
                  subset = diff3 > 0 | is.na(diff3),
                  data=df2_clean)


test.model <- cox.zph(results)
test.model
ggcoxzph(test.model)

?survSplit
model5 <- survSplit (Surv(dateDiff, pos_covid) ~ THIRD_VACCINE_IND +
hourBin + Age2 + HTN_bin + obesity + gender + diabetes_bin + CKD_bin,
                     subset = diff3 > 0 | is.na(diff3), cut = c(250,
350), episode = "timegroup", data=df2_clean)
model5
fit5 <- coxph (Surv(dateDiff, pos_covid) ~
THIRD_VACCINE_IND*strata(timegroup) + hourBin + Age2 + HTN_bin + obesity
+ gender + diabetes_bin + CKD_bin,
               data=model5)
fit5
test.model <- cox.zph(fit5)
test.model


###### Rhythm analysis###
##Analysis for dose 2####
df2 <- read.csv('general2.csv')

df2$hourBin <- case_when((df2$Hour1>=8 & df2$Hour1<=10 & df2$Hour2>=8 &
df2$Hour2<=10) ~ 'Early',
                         (df2$Hour1>=10 & df2$Hour1<=12 & df2$Hour2>=10 &
df2$Hour2<=12) ~ 'Late',
)
df2$hourBin <- factor(df2$hourBin)
df2_clean <- df2 %>% drop_na(hourBin) # total data within the
morning/evening definition is 550163
```

```
df2_clean <- df2_clean %>% filter(FOURTH_VACCINE_IND == "0") ####Analytic
approach number 1 of cox regression and time-dependent variable with
exclusion of second booster###
df2_clean$FOURTH_VACCINE_IND

df2_clean$pos_covid <- case_when((df2_clean$IS_POSITIVE_CD==2 |
df2_clean$IS_POSITIVE_CD==21) ~ 1,
                                 TRUE ~ 0)

summary(as.factor(df2_clean$pos_covid))

baseDate <- as.Date(df2_clean$Date2) + 6
df2_clean$Sample_date_censor <- if_else (is.na(df2_clean$Sample_date),
"2022-04-26",df2_clean$Sample_date)
dateDiff <- as.Date(df2_clean$Sample_date_censor) - baseDate

df2_clean$dateDiff <- as.numeric (dateDiff)

df2_clean <- subset(df2_clean, df2_clean$dateDiff > 0 |
is.na(df2_clean$dateDiff))

sort (df2_clean$Date3)
df2_clean$landmark3 <- as.Date("2021-07-04")  ####Landmark - the first
day for dose 3###
df2_clean$endDate <- as.Date("2022-04-26")
df2_clean$diff3 <- as.Date(df2_clean$Sample_date) -
as.Date(df2_clean$landmark3)
hist (as.numeric(df2_clean$diff3))

df2_clean$diff3 <- as.numeric(df2_clean$diff3)
df2_clean$diff3
df2_clean$THIRD_VACCINE_IND <- as.factor(df2_clean$THIRD_VACCINE_IND)

df2_clean$Age2_old <- case_when(df2_clean$Age2 <= 60 ~ 0,
                                df2_clean$Age2 > 60 ~ 1)

df2_clean$Age2_old <- as.factor(df2_clean$Age2_old)

df2_clean$obesity <- case_when (df2_clean$BMI <= 30 ~ 0,
                                df2_clean$BMI > 30 ~ 1)

df2_clean$obesity <- as.factor(df2_clean$obesity)

df2_clean$diabetes_bin <- case_when(df2_clean$STATUS_IN_DIABETIC == 1 ~
1,
                                    df2_clean$STATUS_IN_DIABETIC == 0 ~
0)


df2_clean$HTN_bin <- case_when(df2_clean$STATUS_IN_BLOODPRESURE == 1 ~ 1,
                               df2_clean$STATUS_IN_BLOODPRESURE == 0 ~ 0)

df2_clean$CKD_bin <- case_when(df2_clean$STATUS_IN_CKD == 1 ~ 1,
```

```
                              df2_clean$STATUS_IN_CKD == 0 ~ 0)

df2_clean$gender <- as.factor(df2_clean$CUSTOMER_SEX_CODE)

df2_clean$Age2_old <- as.factor(df2_clean$Age2_old)
df2_clean$STATUS_IN_DIABETIC <- as.factor(df2_clean$STATUS_IN_DIABETIC)
df2_clean$diabetes_bin <- as.factor(df2_clean$diabetes_bin)
df2_clean$obesity <- as.factor(df2_clean$obesity)
df2_clean$HTN_bin <- as.factor(df2_clean$HTN_bin)
df2_clean$CKD_bin <- as.factor(df2_clean$CKD_bin)
df2_clean$immunosup_bin <- as.factor(df2_clean$immunosup_bin)


x1 <- coxph (Surv(dateDiff, pos_covid) ~ THIRD_VACCINE_IND + hourBin +
Age2_old + obesity + gender + diabetes_bin + CKD_bin,
             subset = diff3 > 0 | is.na(diff3),
             data=df2_clean) %>%
  gtsummary::tbl_regression (exp=TRUE)

x1

#####Similar code was built for each hours bin in interval of two
hours#####
###Schoenfeld test for cox assumption###


schoenfeld <- coxph (Surv(dateDiff, pos_covid) ~ THIRD_VACCINE_IND +
gender + hourBin + diabetes_bin + Age2_old + obesity + CKD_bin +HTN_bin,
                     subset = diff3 > 0 | is.na(diff3),
                     data=df2_clean)

schoenfeld

test.model <- cox.zph(schoenfeld)
test.model


schoenfeld <- ggcoxzph(test.model)
schoenfeld

postscript('schoenfeld.eps')
dev.off()

setEPS()
schoenfeld3 <- ggcoxzph(test.model)
cairo_ps(file='schoenfeld2.jpeg')
plot(1,10)
dev.off()

ggsave (file='schoenfeld3.eps', scale = 1, limitsize = FALSE)


###Sanity test with bootstrap analysis (see mwthods section#####
library(boot)
```

```
boot.cox <- function(df, indices) {
  samples <- df[indices, ]
  fit <-  coxph (Surv(dateDiff, pos_covid) ~ THIRD_VACCINE_IND + hourBin
+ gender + Age2_old + obesity + diabetes_bin + CKD_bin,
                  subset = diff3 > 0 | is.na(diff3),
                  data=samples)
  coef(fit)
}

model.boot <- boot(df2_clean, boot.cox, 2000, parallel = 'snow')
#boot.ci(model.boot, index=2)
model.boot$t0
# grab hourBIN (t0=2)
quantile(model.boot$t[,2],probs = c(0.025, 0.0975))


#####rhythm analysis for dose 3######

df3 <- read.csv('general3.csv')

df3$hourBin <- case_when((df3$Hour3>=8 & df3$Hour3<=10) ~ 'Early',
                         (df3$Hour3>=16 & df3$Hour3<=19) ~ 'Late',
)
df3$hourBin
df3$hourBin <- factor(df3$hourBin)
df3_clean <- df3 %>% drop_na(hourBin) # total data within the
morning/evening definition is 550163

df3_clean$pos_covid <- case_when((df3_clean$IS_POSITIVE_CD==2 |
df3_clean$IS_POSITIVE_CD==21) ~ 1,
                                 TRUE ~ 0
)
summary(as.factor(df3_clean$pos_covid))

baseDate <- as.Date(df3_clean$Date2) + 6
df3_clean$Sample_date_censor <- if_else (is.na(df3_clean$Sample_date),
"2022-04-26",df3_clean$Sample_date)
dateDiff <- as.Date(df3_clean$Sample_date_censor) - baseDate
hist(as.numeric(dateDiff))

df3_clean$dateDiff <- as.numeric (dateDiff)

df3_clean <- subset(df3_clean, df3_clean$dateDiff > 0 |
is.na(df3_clean$dateDiff))

sort (df3_clean$Date4)
df3_clean$landmark4 <- as.Date("2021-09-21")   #The first day of the
fourth vaccine as the landmark date####
df3_clean$endDate <- as.Date("2022-04-26")
df3_clean$diff4 <- as.Date(df3_clean$Sample_date) -
as.Date(df3_clean$landmark4)
hist (as.numeric(df3_clean$diff4))

df3_clean$diff4 <- as.numeric(df3_clean$diff4)
```

```
df3_clean$diff4
df3_clean$FOURTH_VACCINE_IND <- as.factor(df3_clean$FOURTH_VACCINE_IND)

df3_clean$Age3_old <- case_when(df3_clean$Age3 <= 60 ~ 0,
                                df3_clean$Age3 > 60 ~ 1)

df3_clean$Age3_old <- as.factor(df3_clean$Age3_old)

when (df3_clean$BMI <= 30 ~ 0,
                                df3_clean$BMI > 30 ~ 1)

df3_clean$obesity <- as.factor(df3_clean$obesity)


df3_clean$diabetes_bin <- case_when(df3_clean$STATUS_IN_DIABETIC == 1 ~ 
1,
                                    df3_clean$STATUS_IN_DIABETIC == 0 ~ 
0)


df3_clean$HTN_bin <- case_when(df3_clean$STATUS_IN_BLOODPRESURE == 1 ~ 1,
                               df3_clean$STATUS_IN_BLOODPRESURE == 0 ~ 0)

df3_clean$CKD_bin <- case_when(df3_clean$STATUS_IN_CKD == 1 ~ 1,

                               df3_clean$STATUS_IN_CKD == 0 ~ 0)


df3_clean$immunosup_bin <- case_when(df3_clean$IMMUNOSUPPRESSION_Status 
== 1 ~ 1,

                                     df3_clean$IMMUNOSUPPRESSION_Status 
== 0 ~ 0)

df3_clean$SES_bin <- as.factor(df3_clean$SES_bin)
df3_clean$Age3_old <- as.factor(df3_clean$Age3_old)
df3_clean$STATUS_IN_DIABETIC <- as.factor(df3_clean$STATUS_IN_DIABETIC)
df3_clean$diabetes_bin <- as.factor(df3_clean$diabetes_bin)
df3_clean$obesity <- as.factor(df3_clean$obesity)
df3_clean$HTN_bin <- as.factor(df3_clean$HTN_bin)
df3_clean$CKD_bin <- as.factor(df3_clean$CKD_bin)
df3_clean$immunosup_bin <- as.factor(df3_clean$immunosup_bin)

model_dose3_infections <- coxph (Surv(dateDiff, pos_covid) ~ 
FOURTH_VACCINE_IND + hourBin + Age3_old + obesity + gender + diabetes_bin 
+ HTN_bin + CKD_bin,
                                 subset = diff4 > 0 | is.na(diff4),
                                 data=df3_clean) %>%
  gtsummary::tbl_regression (exp=TRUE)

model_dose3_infections

###Similar approach was used in bins of 2 hours while 8am-10am is the 
anchor (see methods)####
```

```
###Rhythm analysis for dose 4######

df4 <- read.csv('general4.csv')

df4$hourBin <- case_when((df4$Hour4>=8 & df4$Hour4<=10) ~ 'Early',
                         (df4$Hour4>=16 & df4$Hour4<=19) ~ 'Late',
)
df4$hourBin
df4$hourBin <- factor(df4$hourBin)
df4_clean <- df4 %>% drop_na(hourBin) # total data within the
morning/evening definition is 550163

df4_clean$pos_covid <- case_when((df4_clean$IS_POSITIVE_CD==2 |
df4_clean$IS_POSITIVE_CD==21) ~ 1,
                                 TRUE ~ 0
)
summary(as.factor(df4_clean$pos_covid))


baseDate <- as.Date(df4_clean$Date4) + 6

df4_clean$sample_date_censor <- if_else (is.na(df4_clean$Sample_date),
"2022-04-26",df4_clean$Sample_date)

dateDiff <- as.Date(df4_clean$sample_date_censor) - baseDate

hist(as.numeric(dateDiff))

df4_clean$dateDiff <- as.numeric(dateDiff)

df4_clean <- subset(df4_clean, df4_clean$dateDiff > 0 |
is.na(df4_clean$dateDiff))

df4_clean$Age4_old <- case_when(df4_clean$Age4 <= 60 ~ 0,
                                df4_clean$Age4 > 60 ~ 1)

df4_clean$endDate <- "2022-04-26"



# cox without time variable (i.e. not long format)
df4_clean$dateDiff <- as.numeric( df4_clean$dateDiff)

df4_clean$obesity <- case_when (df4_clean$BMI <= 30 ~ 0,
                                df4_clean$BMI > 30 ~ 1)

df4_clean$obesity <- as.factor(df4_clean$obesity)

df4_clean$diabetes_bin <- case_when(df4_clean$STATUS_IN_DIABETIC == 1 ~
1,
                                    df4_clean$STATUS_IN_DIABETIC == 0 ~
0)
```

```
df4_clean$HTN_bin <- case_when(df4_clean$STATUS_IN_BLOODPRESURE == 1 ~ 1,
                               df4_clean$STATUS_IN_BLOODPRESURE == 0 ~ 0)

df4_clean$CKD_bin <- case_when(df4_clean$STATUS_IN_CKD == 1 ~ 1,

                               df4_clean$STATUS_IN_CKD == 0 ~ 0)


df4_clean$immunosup_bin <- case_when(df4_clean$IMMUNOSUPPRESSION_Status
== 1 ~ 1,

                                     df4_clean$IMMUNOSUPPRESSION_Status
== 0 ~ 0)

df4_clean$SES_bin <- as.factor(df4_clean$SES_bin)
df4_clean$Age4_old <- as.factor(df4_clean$Age4_old)
df4_clean$STATUS_IN_DIABETIC <- as.factor(df4_clean$STATUS_IN_DIABETIC)
df4_clean$diabetes_bin <- as.factor(df4_clean$diabetes_bin)
df4_clean$obesity <- as.factor(df4_clean$obesity)
df4_clean$HTN_bin <- as.factor(df4_clean$HTN_bin)
df4_clean$CKD_bin <- as.factor(df4_clean$CKD_bin)
df4_clean$immunosup_bin <- as.factor(df4_clean$immunosup_bin)

model2 <- coxph(
  Surv(time = df4_clean$dateDiff, event = df4_clean$pos_covid) ~ hourBin
+ Age4_old + obesity + gender + diabetes_bin + HTN_bin + CKD_bin
  ,   data = df4_clean)

ggforest(model2)


###Again, similar approach was used for every 2 hours bin while 8am-10am
was the anchor####


####Validation of rhytmic analysis by casual inference######

library(tidyverse)
library(dplyr)
library(tidyr)

df <- read.csv('general2.csv')


## mutate to add time to dose (3 and 4)
df <- df[,3:97]

df2 <- df %>% mutate(
  t0 = Date2,
  diff2_3 = as.Date(Date3) - as.Date(t0),
  diff2_4 =as.Date(Date4) - as.Date(t0)
)
```

```
df2$hourBin <- case_when((df2$Hour2>=8 & df2$Hour2<=11) ~ 'Morning',
                         (df2$Hour2>=16 & df2$Hour2<=19) ~ 'Evening',
                         (df2$Hour2>=12 & df2$Hour2<=16) ~ 'Mid'
)

df2$pos_covid <- case_when((df2$IS_POSITIVE_CD==2 |
df2$IS_POSITIVE_CD==21) ~ 1,
                           TRUE ~ 0
)

# censoring people who received dose 4
# first set a variable 0 if date 4 is before the COVID test and 1×‟ if
its after
df2$isDat4 <- case_when(as.Date(df2$Date4) - as.Date(df2$Sample_date) < 0
~ 1,
                        TRUE ~ 0
                        #as.Date(df2$Date4) - as.Date(df2$Sample_date) <0
~ 0
)

#df2 %>% select(Date4, isDat4, Sample_date)

summary(as.factor(df2$isDat4))
hist(df2$isDat4)
df2$pos_covid <- case_when((df2$IS_POSITIVE_CD==2 |
df2$IS_POSITIVE_CD==21 & df2$isDat4==0) ~ 1,

                           TRUE ~ 0
)

df2$endDate <- "2022-04-26"
df2$follow_up <- as.Date(df2$endDate) - as.Date(df2$Date2)

df2$pos_covid <- as.factor(df2$pos_covid)



cleanDat <- function(df2) {
  df2_clean <- df2 %>% drop_na(hourBin)

  df2_clean$Age2_old <- case_when(df2_clean$Age2 <= 60 ~ 0,
                                  df2_clean$Age2 > 60 ~ 1)

  df2_clean$diabetes_bin <- case_when(df2_clean$STATUS_IN_DIABETIC == 1 ~
1,
                                      df2_clean$STATUS_IN_DIABETIC == 0 ~
0)


  df2_clean$HTN_bin <- case_when(df2_clean$STATUS_IN_BLOODPRESURE == 1 ~
1,
```

```
                                  df2_clean$STATUS_IN_BLOODPRESURE == 0 ~
0)

  df2_clean$CKD_bin <- case_when(df2_clean$STATUS_IN_CKD == 1 ~ 1,

                                  df2_clean$STATUS_IN_CKD == 0 ~ 0)


  df2_clean$immunosup_bin <- case_when(df2_clean$IMMUNOSUPPRESSION_Status
== 1 ~ 1,

                                        df2_clean$IMMUNOSUPPRESSION_Status
== 0 ~ 0)


  df2_clean$dialisis_bin <- case_when(df2_clean$STATUS_IN_DIALIZA==1 ~ 1,
                                       df2_clean$STATUS_IN_DIALIZA==0 ~ 0)

  df2_clean$astma <- case_when(df2_clean$Asthma_ever == 1 ~ 1,
                                df2_clean$Asthma_ever == 0 ~ 0)

  df2_clean$nerologic <- case_when(df2_clean$Neurologic_disease_ever == 1
~ 1,
                                    df2_clean$Neurologic_disease_ever == 0
~ 0)

  df2_clean$obesity <- case_when (df2_clean$BMI <= 30 ~ 0,
                                   df2_clean$BMI > 30 ~ 1)


  # set as factors

  df2_clean$Age2_old <- as.factor(df2_clean$Age2_old)
  df2_clean$STATUS_IN_DIABETIC <- as.factor(df2_clean$STATUS_IN_DIABETIC)
  df2_clean$diabetes_bin <- as.factor(df2_clean$diabetes_bin)
  df2_clean$obesity <- as.factor(df2_clean$obesity)
  df2_clean$HTN_bin <- as.factor(df2_clean$HTN_bin)
  df2_clean$CKD_bin <- as.factor(df2_clean$CKD_bin)
  df2_clean$immunosup_bin <- as.factor(df2_clean$immunosup_bin)
  df2_clean$gender <- as.factor(df2_clean$CUSTOMER_SEX_CODE)

  return(df2_clean)
}

# change to long format (each subject has three lines (dose 2,3,4))
#df_long <- df2_clean %>% gather(cond_diff, diff, diff2_3:diff2_4)



### Univariate Survival analysis
library(survival)
library(survminer)
library(ggfortify)
```

```
model1 <- coxph (Surv(follow_up, pos_covid) ~  gender + hourBin +
                   diabetes_bin + Age2_old + obesity + CKD_bin +HTN_bin
+diff2_3,
                 cluster = InternalPatID, id = InternalPatID,
                 data=df2_clean)

summary(model1)

model1 %>% gtsummary::tbl_regression(exp=T)


# test permutation of all options of hourbin (i.e., Am:PM, AM:AM etc/)

# generate specific hourBin
df2$hourBinP <- case_when((df2$Hour1>=8 & df2$Hour1<=11 & df2$Hour2>=8 &
df2$Hour2<=11) ~ 'AMAM',
                          (df2$Hour1>=8 & df2$Hour1<=11 & df2$Hour2>=16 &
df2$Hour2<=19) ~ 'AMPM',
                          (df2$Hour1>=16 & df2$Hour1<=19 & df2$Hour2>=8 &
df2$Hour2<=11) ~ 'PMAM',
                          (df2$Hour1>=16 & df2$Hour1<=19 & df2$Hour2>=16
& df2$Hour2<=19) ~ 'PMPM'
)


model2 <- coxph (Surv(follow_up, pos_covid) ~  gender + hourBinP +
                   diabetes_bin + Age2_old + obesity + CKD_bin +HTN_bin
+diff2_3,
                 cluster = InternalPatID, id = InternalPatID,
                 data=df2_clean)

summary(model2)
model2 %>% gtsummary::tbl_regression(exp=T)


# compare morning to evening only
df2$hourBin <- case_when((df2$Hour2>=8 & df2$Hour2<=11) ~ 'AM',
                         (df2$Hour2>=16 & df2$Hour2<=19) ~ 'PM',

)

# go back to run all data cleaning and factorization
df2_cleanAMPM <- cleanDat(df2) # clean data

model3 <- coxph (Surv(follow_up, pos_covid) ~  gender + hourBinP +
                   diabetes_bin + Age2_old + obesity + CKD_bin +HTN_bin
+diff2_3,
                 cluster = InternalPatID, id = InternalPatID,
                 data=df2_cleanAMPM)

summary(model3)
model3 %>% gtsummary::tbl_regression(exp=T)
```

```
###COVID-related ER visit analysis###

library(survival)
library(survminer)
library(ggfortify)
library(tidyverse)

df2 <- read.csv('general2.csv')

df2$hourBin <- case_when((df2$Hour2>=8 & df2$Hour2<=11) ~ 'Morning',
                         (df2$Hour2>=16 & df2$Hour2<=19) ~ 'Evening',
)

df2 <- df2 %>% mutate(
  t0 = Date2,
  diff2_3 = as.Date(Date3) - as.Date(t0),
  diff2_4 =as.Date(Date4) - as.Date(t0)
)

df2$hourBin <- factor(df2$hourBin)
# add censoring of Dose 4
# censoring people who received dose 4
# first set a variable 0 if date 4 is before the ER visit
df2$isDat4 <- case_when(as.Date(df2$Date4) - as.Date(df2$ER_VISIT) < 0 ~
1,
                        TRUE ~ 0

)
# create new variable er for visit after censoring dose4
df2$er <- case_when((df2$ER_Visit_indicator==1 & df2$isDat4==0) ~ 1,

                    TRUE ~ 0
)


df2_clean <- df2 %>% drop_na(hourBin) # total data within the
morning/evening definition is 550163

#df2_clean <- df2_clean %>% filter(FOURTH_VACCINE_IND == "0")
df2_clean$er <- as.factor(df2_clean$er)
# add censoring of Dose 4


summary(df2_clean$er)
baseDate <- as.Date(df2_clean$Date2) + 6
df2_clean$ER_VISIT_censor <- if_else (is.na(df2_clean$ER_VISIT), "2022-
04-26",df2_clean$ER_VISIT)
dateDiff <- as.Date(df2_clean$ER_VISIT_censor) - baseDate
hist(as.numeric(dateDiff))

df2_clean$dateDiff <- as.numeric (dateDiff)
```

```
df2_clean <- subset(df2_clean, df2_clean$dateDiff > 0 |
is.na(df2_clean$dateDiff))


model2 <- coxph (Surv(dateDiff, er) ~ diff2_3 + hourBin,
                 cluster=InternalPatID, id = InternalPatID,
                 data=df2_clean) %>%
  gtsummary::tbl_regression (exp=TRUE)

model2


# add all other relevant vaiables
df2_clean$Age2_old <- case_when(df2_clean$Age2 <= 60 ~ 0,
                                df2_clean$Age2 > 60 ~ 1)

df2_clean$diabetes_bin <- case_when(df2_clean$STATUS_IN_DIABETIC == 1 ~
1,
                                    df2_clean$STATUS_IN_DIABETIC == 0 ~
0)


df2_clean$HTN_bin <- case_when(df2_clean$STATUS_IN_BLOODPRESURE == 1 ~ 1,
                               df2_clean$STATUS_IN_BLOODPRESURE == 0 ~ 0)

df2_clean$CKD_bin <- case_when(df2_clean$STATUS_IN_CKD == 1 ~ 1,

                               df2_clean$STATUS_IN_CKD == 0 ~ 0)


df2_clean$immunosup_bin <- case_when(df2_clean$IMMUNOSUPPRESSION_Status
== 1 ~ 1,

                                     df2_clean$IMMUNOSUPPRESSION_Status
== 0 ~ 0)


df2_clean$dialisis_bin <- case_when(df2_clean$STATUS_IN_DIALIZA==1 ~ 1,
                                    df2_clean$STATUS_IN_DIALIZA==0 ~ 0)

df2_clean$astma <- case_when(df2_clean$Asthma_ever == 1 ~ 1,
                             df2_clean$Asthma_ever == 0 ~ 0)

df2_clean$nerologic <- case_when(df2_clean$Neurologic_disease_ever == 1 ~
1,
                                 df2_clean$Neurologic_disease_ever == 0 ~
0)

df2_clean$obesity <- case_when (df2_clean$BMI <= 30 ~ 0,
                                df2_clean$BMI > 30 ~ 1)


# set as factors
```

```
df2_clean$Age2_old <- as.factor(df2_clean$Age2_old)
df2_clean$STATUS_IN_DIABETIC <- as.factor(df2_clean$STATUS_IN_DIABETIC)
df2_clean$diabetes_bin <- as.factor(df2_clean$diabetes_bin)
df2_clean$obesity <- as.factor(df2_clean$obesity)
df2_clean$HTN_bin <- as.factor(df2_clean$HTN_bin)
df2_clean$CKD_bin <- as.factor(df2_clean$CKD_bin)
df2_clean$immunosup_bin <- as.factor(df2_clean$immunosup_bin)
df2_clean$gender <- as.factor(df2_clean$CUSTOMER_SEX_CODE)


model3 <-coxph (Surv(dateDiff, er) ~  gender + hourBin +
                  diabetes_bin + Age2_old + obesity + CKD_bin +HTN_bin
+diff2_3,
                cluster = InternalPatID, id = InternalPatID,
                data=df2_clean)

model3 %>% gtsummary::tbl_regression (exp=TRUE)


### Sinus function for ER

sinusBin2 <- function(df, howrCompare, hourBase) {

  # df is the datframe, howrcompare hours to compare (a list of vectors
sized 2). hourBase - baseline, vector sized 2
  # ifage - include or not age in the analysis (1 = include, 0 = not)
  df$hourBin <- case_when((df$Hour2>=hourBase[1] & df$Hour2<hourBase[2])
~ 'Early',
                          (df$Hour2>=howrCompare[1] &
df$Hour2<howrCompare[2]) ~ 'Late',
  )

  df$hourBin <- factor(df$hourBin, levels = c("Early","Late"))

  df2_clean <- df %>% drop_na(hourBin)

  df2_clean$Age2_old <- case_when(df2_clean$Age2 <= 60 ~ 0,
                                  df2_clean$Age2 > 60 ~ 1)

  df2_clean$diabetes_bin <- case_when(df2_clean$STATUS_IN_DIABETIC == 1 ~
1,
                                      df2_clean$STATUS_IN_DIABETIC == 0 ~
0)


  df2_clean$HTN_bin <- case_when(df2_clean$STATUS_IN_BLOODPRESURE == 1 ~
1,
                                 df2_clean$STATUS_IN_BLOODPRESURE == 0 ~
0)

  df2_clean$CKD_bin <- case_when(df2_clean$STATUS_IN_CKD == 1 ~ 1,

                                 df2_clean$STATUS_IN_CKD == 0 ~ 0)
```

```
  df2_clean$immunosup_bin <- case_when(df2_clean$IMMUNOSUPPRESSION_Status
== 1 ~ 1,

                                       df2_clean$IMMUNOSUPPRESSION_Status
== 0 ~ 0)


  df2_clean$dialisis_bin <- case_when(df2_clean$STATUS_IN_DIALIZA==1 ~ 1,
                                      df2_clean$STATUS_IN_DIALIZA==0 ~ 0)

  df2_clean$astma <- case_when(df2_clean$Asthma_ever == 1 ~ 1,
                               df2_clean$Asthma_ever == 0 ~ 0)

  df2_clean$nerologic <- case_when(df2_clean$Neurologic_disease_ever == 1
~ 1,
                                   df2_clean$Neurologic_disease_ever == 0
~ 0)

  df2_clean$obesity <- case_when (df2_clean$BMI <= 30 ~ 0,
                                  df2_clean$BMI > 30 ~ 1)


  # set as factors

  df2_clean$Age2_old <- as.factor(df2_clean$Age2_old)
  df2_clean$STATUS_IN_DIABETIC <- as.factor(df2_clean$STATUS_IN_DIABETIC)
  df2_clean$diabetes_bin <- as.factor(df2_clean$diabetes_bin)
  df2_clean$obesity <- as.factor(df2_clean$obesity)
  df2_clean$HTN_bin <- as.factor(df2_clean$HTN_bin)
  df2_clean$CKD_bin <- as.factor(df2_clean$CKD_bin)
  df2_clean$immunosup_bin <- as.factor(df2_clean$immunosup_bin)
  df2_clean$gender <- as.factor(df2_clean$CUSTOMER_SEX_CODE)

  baseDate <- as.Date(df2_clean$Date2) + 6
  df2_clean$ER_VISIT_censor <- if_else (is.na(df2_clean$ER_VISIT), "2022-
04-26",df2_clean$ER_VISIT)
  dateDiff <- as.Date(df2_clean$ER_VISIT_censor) - baseDate
  df2_clean$dateDiff <- as.numeric (dateDiff)



  model2 <-coxph(Surv(dateDiff, er) ~  gender + hourBin +
                   diabetes_bin + Age2_old + obesity + CKD_bin +HTN_bin
+diff2_3,
                 cluster = InternalPatID, id = InternalPatID,
                 data=df2_clean)

  a <- print(summary(df2_clean$hourBin))

  return(list(model2, a))

}
```

```
## 2 hours bin
hourBase <- c(8,10)
hours <- list(c(10,12),c(11,13), c(12,14), c(13,15), c(14,16), c(15,17),
c(16,18), c(17,19), c(18,20),
              c(19,21), c(20,22), c(21,23))

result <- matrix(nrow = length(hours), ncol=5)
colnames(result) <- c('hour','HR','lower CI', 'higher CI', "N")
for (i in 1:length(hours)){
  model1 <- sinusBin2(df = df2, howrCompare = hours[[i]], hourBase =
hourBase)
  result[i,1] <- paste(hours[[i]],collapse = '_')
  result[i,2] <- summary(model1[[1]])$conf.int[2,1]
  result[i,3] <- summary(model1[[1]])$conf.int[2,3]
  result[i,4] <- summary(model1[[1]])$conf.int[2,4]
  result[i,5] <- model1[[2]][2]
}


write.csv(result, file="Dose2_2hrsBinMovingAverage_allAges_ED.csv")

####similar analytic approach was used for COVID-related ER visits post
doses 3, 4###

##Admissions analysis###
df2$hourBin <- case_when((df2$Hour1>=8 & df2$Hour1<=10 & df2$Hour2>=8 &
df2$Hour2<=10) ~ 'Early',
                         (df2$Hour1>=10 & df2$Hour1<=12 & df2$Hour2>=10 &
df2$Hour2<=12) ~ 'Late',
)
df2$hourBin
df2$hourBin <- factor(df2$hourBin)
df2_clean <- df2 %>% drop_na(hourBin) # total data within the
morning/evening definition is 550163

df2_clean <- df2_clean %>% filter(FOURTH_VACCINE_IND == "0")
df2_clean$admission_indicator


summary(df2_clean$admission_indicator)
baseDate <- as.Date(df2_clean$Date2) + 6
df2_clean$first_admission_censor <- if_else
(is.na(df2_clean$first_admission), "2022-04-
26",df2_clean$first_admission)
dateDiff <- as.Date(df2_clean$first_admission_censor) - baseDate

df2_clean$dateDiff <- as.numeric (dateDiff)

df2_clean <- subset(df2_clean, df2_clean$dateDiff > 0 |
is.na(df2_clean$dateDiff))

df2_clean$landmark3 <- as.Date("2021-07-04")
df2_clean$endDate <- as.Date("2022-04-26")
```

```
df2_clean$diff3 <- as.Date(df2_clean$first_admission) -
as.Date(df2_clean$landmark3)

df2_clean$diff3 <- as.numeric(df2_clean$diff3)
df2_clean$diff3
df2_clean$THIRD_VACCINE_IND <- as.factor(df2_clean$THIRD_VACCINE_IND)
df2_clean$admission_indicator

df2_clean$Age2_old <- case_when(df2_clean$Age2 <= 60 ~ 0,
                                df2_clean$Age2 > 60 ~ 1)

df2_clean$obesity <- case_when (df2_clean$BMI <= 30 ~ 0,
                                df2_clean$BMI > 30 ~ 1)

df2_clean$diabetes_bin <- case_when(df2_clean$STATUS_IN_DIABETIC == 1 ~
1,
                                    df2_clean$STATUS_IN_DIABETIC == 0 ~
0)


df2_clean$HTN_bin <- case_when(df2_clean$STATUS_IN_BLOODPRESURE == 1 ~ 1,
                               df2_clean$STATUS_IN_BLOODPRESURE == 0 ~ 0)

df2_clean$CKD_bin <- case_when(df2_clean$STATUS_IN_CKD == 1 ~ 1,

                               df2_clean$STATUS_IN_CKD == 0 ~ 0)


df2_clean$immunosup_bin <- case_when(df2_clean$IMMUNOSUPPRESSION_Status
== 1 ~ 1,

                                     df2_clean$IMMUNOSUPPRESSION_Status
== 0 ~ 0)

df2_clean$Age2_old <- as.factor(df2_clean$Age2_old)
df2_clean$STATUS_IN_DIABETIC <- as.factor(df2_clean$STATUS_IN_DIABETIC)
df2_clean$diabetes_bin <- as.factor(df2_clean$diabetes_bin)
df2_clean$obesity <- as.factor(df2_clean$obesity)
df2_clean$HTN_bin <- as.factor(df2_clean$HTN_bin)
df2_clean$CKD_bin <- as.factor(df2_clean$CKD_bin)
df2_clean$immunosup_bin <- as.factor(df2_clean$immunosup_bin)
df2_clean$gender <- as.factor(df2_clean$CUSTOMER_SEX_CODE)

x1 <- coxph (Surv(dateDiff, admission_indicator) ~ THIRD_VACCINE_IND +
hourBin + Age2_old + gender + obesity + diabetes_bin + HTN_bin + CKD_bin,
             subset = diff3 > 0 | is.na(diff3),
             data=df2_clean) %>%
  gtsummary::tbl_regression (exp=TRUE)

x1

####Similar analytic approach was used in 2 hours bins, while 8am-10am is
the anchor. Also, similar code was used for doses 3, 4 as above#
```
